# Moth‐Wing‐Inspired Multifunctional Metamaterials

**DOI:** 10.1002/adma.202515350

**Published:** 2025-12-16

**Authors:** Haoran Pei, Hang Yang, Ning Zhang, Tian Li, Xinxin Wang, Miao Zhao, Shuwei Ding, Xin Wang, Qinniu Lv, Zijie Xu, Yinghong Chen, Xinwei Li, Wei Zhai

**Affiliations:** ^1^ State Key Laboratory of Advanced Polymer Materials Polymer Research Institute of Sichuan University Chengdu 610065 China; ^2^ Department of Mechanical Engineering National University of Singapore Singapore 117575 Singapore; ^3^ Newcastle University in Singapore Faculty of Science, Agriculture, and Engineering Newcastle University Newcastle upon Tyne NE1 7RU UK; ^4^ School of Mechanical and Electrical Engineering University of Electronic Science and Technology of China Chengdu 611731 China

**Keywords:** bioinspired design, broadband sound absorption, mechanical energy dissipation, multifunctional metamaterial, thermal insulation

## Abstract

To evade ultrasonic predation by bats, moths have evolved wing scale architectures capable of absorbing and scattering high‐frequency acoustic signals. Drawing inspiration from this natural defense strategy, a bioinspired multifunctional metamaterial is presented that integrates broadband sound absorption, thermal insulation, and mechanical energy dissipation within a unified structural framework. Inspired by the graded pore architecture of moth scales, acoustic performance is first optimized via genetic algorithm–driven pore design and the structures using 3D printing. The resulting metamaterial exhibits broadband acoustic absorption with an average coefficient of 0.742 across the 1000–6000 Hz frequency range. When implemented in helmet‐based noise reduction systems, the proposed metamaterial outperforms conventional commercial foams in suppressing environmental noise. In addition, the metamaterial retains a negative Poisson's ratio under large deformation, which enhances its mechanical energy dissipation and impact resilience. Furthermore, the alternating architecture of polymer layers and internal air cavities reduces the effective thermal conductivity to 30.2 mW m^−1^ K^−1^, ensuring excellent thermal insulation. This work demonstrates that leveraging biological architectures enables the simultaneous integration of acoustic, mechanical, and thermal functionalities in lightweight metamaterials, offering a new paradigm for multifunctional design.

## Graphical abstract

This study develops a moth‐wing‐inspired heterogeneous metamaterial that achieves synergistic broadband sound absorption, mechanical energy dissipation, and thermal insulation within a lightweight architected framework. Combining bioinspired gradient design, genetic‐algorithm optimization, and additive manufacturing, the bionic heterogeneous acoustic metamaterial demonstrates superior multifunctional integration and applicability in advanced noise‐control systems such as protective helmets.

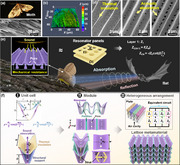

## 1 Introduction

Biologically inspired and biomimetic design strategies for architected materials offer powerful solutions to many of the pressing challenges faced by modern society, driving substantial advances across diverse scientific and engineering domains. The advent of additive manufacturing has further expanded the design space for developing multifunctional biomimetic structural materials at the macroscale, enabling the fabrication of complex 3D architectures through computer‐aided design and freeform manufacturing techniques.^[^ ^1^, ^2^, ^3^ ^]^ Natural biomaterials often possess highly sophisticated heterogeneous structures,^[^ ^4^ ^]^ exemplified by the brick‐and‐mortar configuration of nacre (which imparts both strength and toughness),^[^ ^5^ ^]^ the helicoidal arrangement in mantis shrimp cuticles,^[^ ^6^ ^]^ and the cross‐lamellar architecture found in conch shells.^[^ ^7^ ^]^ Although conventional biomimetic structural designs have proven effective in enhancing mechanical performance, their predominant focus on single‐functionality poses a significant limitation in scenarios demanding concurrent acoustic, thermal, and mechanical capabilities—requirements that are increasingly critical in contemporary engineering systems.

Noise pollution has become an increasingly pressing issue, posing significant threats to both socioeconomic development and human physical & mental well‐being. These effects are particularly critical in safety‐sensitive environments such as industrial production, military operations, and air traffic control. This has led to a surging demand for lightweight, multifunctional sound‐absorbing materials.^[^ ^8^, ^9^, ^10^ ^]^ Conventional sound‐absorbing materials are predominantly porous or fibrous in nature, comprising interconnected open, semi‐open, or closed pore structures with either regular or irregular geometries.^[^ ^11^, ^12^, ^13^ ^]^ Representative examples include polymeric foams, aerogels, and natural fibers such as cotton and linen. However, these materials often exhibit intrinsic limitations—namely, poor low‐to‐mid‐frequency attenuation, low mechanical robustness, and potential health hazards due to fiber shedding, constraining their effectiveness in rigorous engineering applications (Figure S1, Supporting Information).^[^ ^14^ ^]^ In response, recent research efforts have increasingly focused on emerging microlattice metamaterials.^[^ ^15^, ^16^, ^17^ ^]^ These architected materials represent a paradigm shift by emphasizing structural design over chemical composition, featuring enhanced mechanical robustness and programmable physical responses, thereby enabling the integration of multifunction in a single platform.^[^ ^18^, ^19^ ^]^ Despite recent advances, the current metamaterial designs, typically based on homogeneous truss, shell, or plate architectures, still lack the internal microstructures required for effective viscous energy dissipation and the functional heterogeneity necessary to achieve effective broadband sound absorption alongside simultaneous mechanical and thermal performance.^[^ ^20^ ^]^

**Figure 1:**
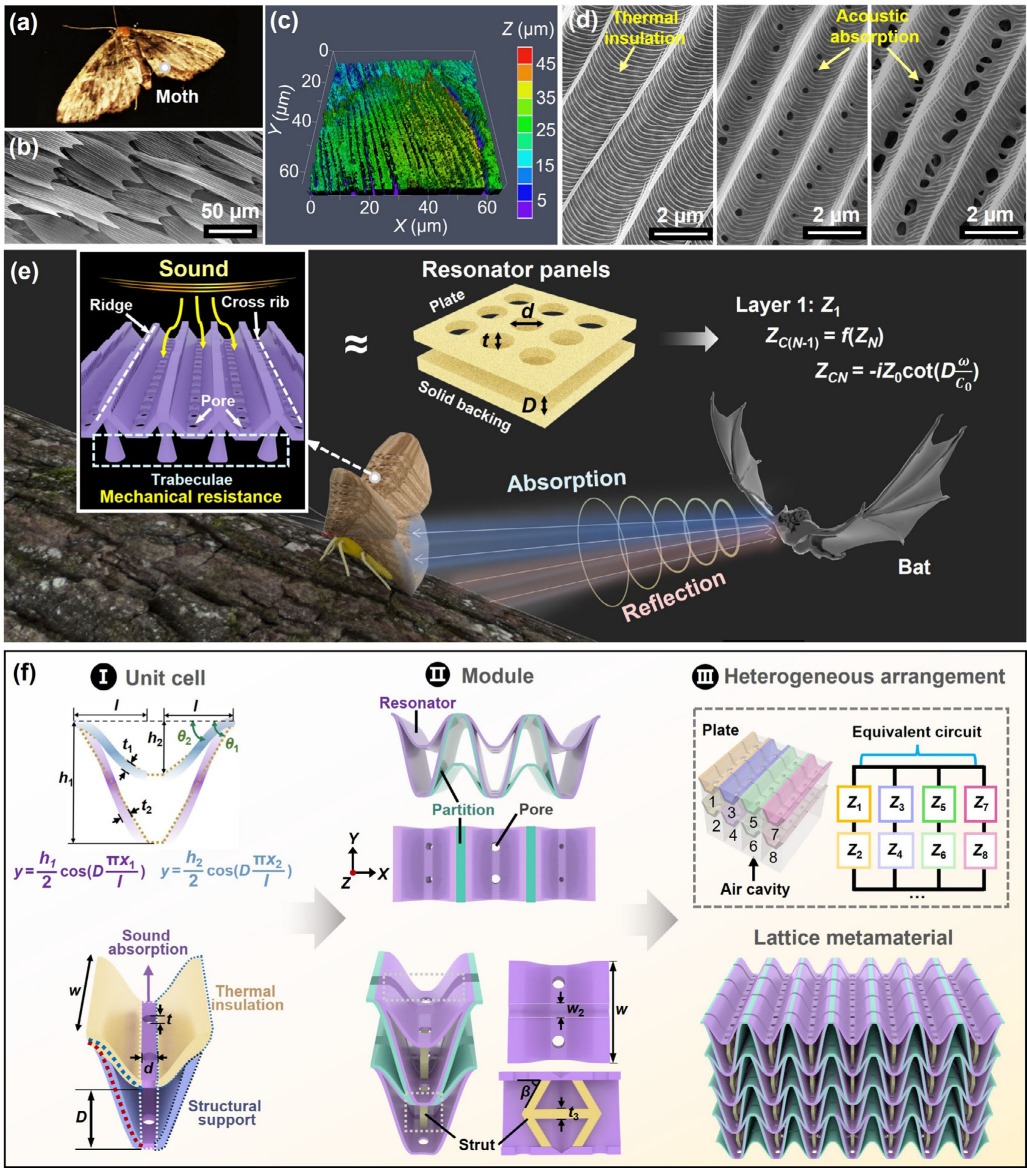
Bioinspired design of a Helmholtz‑type sound‑absorbing metamaterial based on moth wing scales. a) Optical image of a moth. b) SEM micrograph showing the characteristic tile‐like, overlapping arrangement of wing scales. c) LCM image revealing the groove‑and‑ridge microstructure of an individual scale. d) High‑magnification SEM images displaying moth wing scales with varying pores (left to right: none to small and then to large), illustrating combined thermal insulation and broadband acoustic absorption capabilities. e) Schematic diagram of the predator–prey interaction between moths and bats via echolocation, alongside the mechanism of moth wing–inspired perforated acoustic panels. f) Design schematic of the moth wing‐inspired metamaterial, detailing the integration from unit cell to module and periodic lattice with heterogeneous arrangements.

In nature, nocturnal moths engage in a predator–prey relationship with bats, which detect and capture moths using echolocation (**Figure** **1**a). Through prolonged natural evolution and selective pressures, moths have developed a sophisticated, sound‐absorbing protective system to counteract bat predation.^[^ ^21^, ^22^ ^]^ Recent studies have confirmed that moth wings function as acoustic metasurfaces, attenuating the effectiveness of bat sonar by reducing prey detectability.^[^ ^23^ ^]^ However, the underlying mechanisms of this acoustic absorption remain incompletely understood. To investigate this phenomenon, we examined the microstructural features of moth wings. Using a scanning electron microscope (SEM), we observed that these wings are covered in overlapping scales of various morphologies (Figure 1b), and magnified images from a laser confocal microscope (LCM) reveal that the individual scales exhibit multiple parallel grooves (Figure 1c; Figure S2, Supporting Information), which are likely critical to their sound absorption capabilities. Upon further magnification, rows of V‐shaped grooves become apparent (Figure 1d). Similar to those found on butterfly wings, these grooves are reinforced by a network of intersecting ridges and ribs that confer mechanical stability.^[^ ^24^ ^]^ However, despite their morphological similarity, the butterfly wings exhibit an extremely high porosity (Figure S4, Supporting Information), forming ridge–groove photonic structures that generate vivid coloration through light scattering and interference rather than acoustic damping (detailed in Section S2, Supporting Information).^[^ ^25^ ^]^ In contrast, the moth wings employ hierarchically porous scales that induce multiple localized resonances and thermo‐viscous energy losses, serving as an efficient acoustic camouflage against echolocating bats. Even other nocturnal insects such as fireflies lack comparable acoustic adaptations; instead, they rely on chemical defenses and bioluminescent warning signals to deter predators rather than on acoustic stealth.^[^ ^26^ ^]^ Notably, Figure 1d shows a contrast between non‐porous moth wing scales on the left, primarily serving thermal insulation functions, and the graded‐pore scales on the right. We propose that each porous scale can be functionally analogized to a microperforated plate, operating on the Helmholtz resonance principle (Figure 1e). While the wedge‐shaped morphology of the scales may contribute to scattering incident echolocation signals, the perforations within the scales play a more dominant role by enabling the dissipation of acoustic energy through resonance. In this mechanism, incident sound waves interact with air molecules as they pass through the pores and enter the underlying prothorax cavity, where resonant air vibrations dissipate acoustic energy. Through SEM observation of numerous scales (Figure S3, Supporting Information), we found that the smaller pores tend to appear on broader, shorter scales, while larger pores are associated with elongated scales. The graded distribution of pore sizes across the wing surface likely supports multiple resonance modes, thereby enabling broadband sound absorption. Beyond their acoustic function, moth wing scales also provide thermal insulation: the stacked and thickened structure limits heat loss from wing tissues, helping moths conserve metabolic heat and sustain energetically efficient flight.^[^ ^27^ ^]^ This multifunctional biological design thus not only offers a means of acoustic camouflage from echolocating predators but also exemplifies an elegant integration of structural, thermal, and acoustic performance.

Inspired by the multifunctional architecture of moth wings, we propose a bio‐hybrid design strategy that integrates moth wing–inspired heterogeneity with topology optimization enabled by genetic algorithms. The resulting 3D‐printed metamaterial emulates not only the morphological features of moth wing scales but also replicates their cross‐functional performance across acoustic, thermal, and mechanical domains, achieving a level of multifunctional integration unattainable by conventional periodic lattice designs. Heterogeneous sound‐absorbing units were engineered to mimic the graded scale distribution of moth wings, thereby activating multiple resonance modes in concert and significantly broadening the effective acoustic absorption bandwidth. This strategy enabled the development of a broadband absorber with an experimentally measured average sound absorption coefficient of 0.742 over the 1000–6000 Hz frequency range. Moreover, the scale‐inspired architecture exhibits an auxetic behavior under large deformation, which contributes to enhanced mechanical energy dissipation and impact resilience under compressive loading. Simultaneously, the alternating layered configuration of polymer and air, analogous to the multi‐scale thermal insulation strategies observed in moth wings, reduces the effective thermal conductivity to 30.2 mW m^−1^ K^−1^. This integration of broadband sound absorption, mechanical resilience, and thermal insulation within a single lightweight structure represents a new paradigm for bioinspired metamaterials with broad application potential in protective and multifunctional engineering systems.

## 2 Moth‐Wing‐Inspired Metamaterial Design Strategy

We developed a multifunctional metamaterial design strategy inspired by the microarchitecture of moth wings, integrating broadband sound absorption, mechanical energy dissipation, and thermal insulation into a single structural system (Figure 1f). At the unit‐cell level (Figure 1f‐I), the structural framework consists of perforated flat plates interconnected by sinusoidally curved shells. Drawing inspiration from the trabecular support structures observed in moth wing scales, these shells were intentionally designed to exhibit a negative Poisson's ratio, enhancing structural compliance and energy dissipation under mechanical loading. The continuous, smooth geometry serves to minimize manufacturing‐induced defects and mitigate stress concentrations during deformation.^[^ ^28^, ^29^, ^30^ ^]^ Beyond mechanical functionality, thermal insulation was incorporated by mimicking the solid, non‐porous scales on moth wings, which are known to hinder convective and radiative heat loss. To replicate this function, we embedded thermally resistive barriers along the unit walls, utilizing the alternating polymer–air configuration to suppress thermal conductivity.

More critically, the perforations found in moth wing scales are known to contribute significantly to acoustic energy dissipation. Our design leverages this principle through the framework of the multilayer Helmholtz resonator (MLHR) model, an extension of the perforated panel model originally proposed by Maa et al.^[^ ^31^ ^]^ The unit cell geometry is defined in the *x*–*y* plane, where two sinusoidal profiles, *y* = *h*_1_/2cos(*D*π*x*_1_/*l*), *y* = *h*_2_/2cos(*D*π*x*_2_/*l*), are extruded to form cell walls with *t*_1_ = *t*_2_ = 0.5 mm in‐plane thicknesses that prevent cross‐interference and enable independent frequency targeting. By focusing on a multilayer perforated plate architecture, we systematically optimized key geometric parameters, including the pore diameter (*d*), pore depth (*t*), unit width (*w*), and cavity depth (*D*), to enhance both the efficiency and bandwidth of sound absorption. Specifically, by fine‐tuning these dimensions, particularly the *d*, we constructed a heterogeneous parallel‐unit model that mimics the graded scale distribution found in moth wings. This heterogeneity enables the multiple Helmholtz resonance modes to be activated across the array, thereby increasing the average absorption coefficient and extending the effective frequency response range. To ensure high precision in the additive manufacturing process, especially for the small perforations, we implemented hexagonal struts with concave sidewalls (Figure 1f‐II). Such a modular design also introduces additional geometric parameters such as the strut wall thickness (*t*_3_ = 0.5 mm) and the interface angle (*β* = 60°), which represent the wall thickness of the concave hexagonal support and the inclination angle between the hexagonal strut and the panel, respectively. Additionally, a notched connector with a width of *w*_2_ = 1.2 mm was embedded along the perforation axis to provide the local compliance and accommodate deformation under mechanical load. Importantly, fully open gaps were deliberately avoided, as excessive perforation would undermine acoustic performance.

As illustrated in Figure 1f‐III, the final metamaterial is composed of an array of these periodically arranged heterogeneous units, structurally optimized to achieve high broadband sound absorption while preserving mechanical integrity and manufacturability.

## 3 Analytical Acoustics Model

### 3.1 Homogeneous Absorber

**Figure 2:**
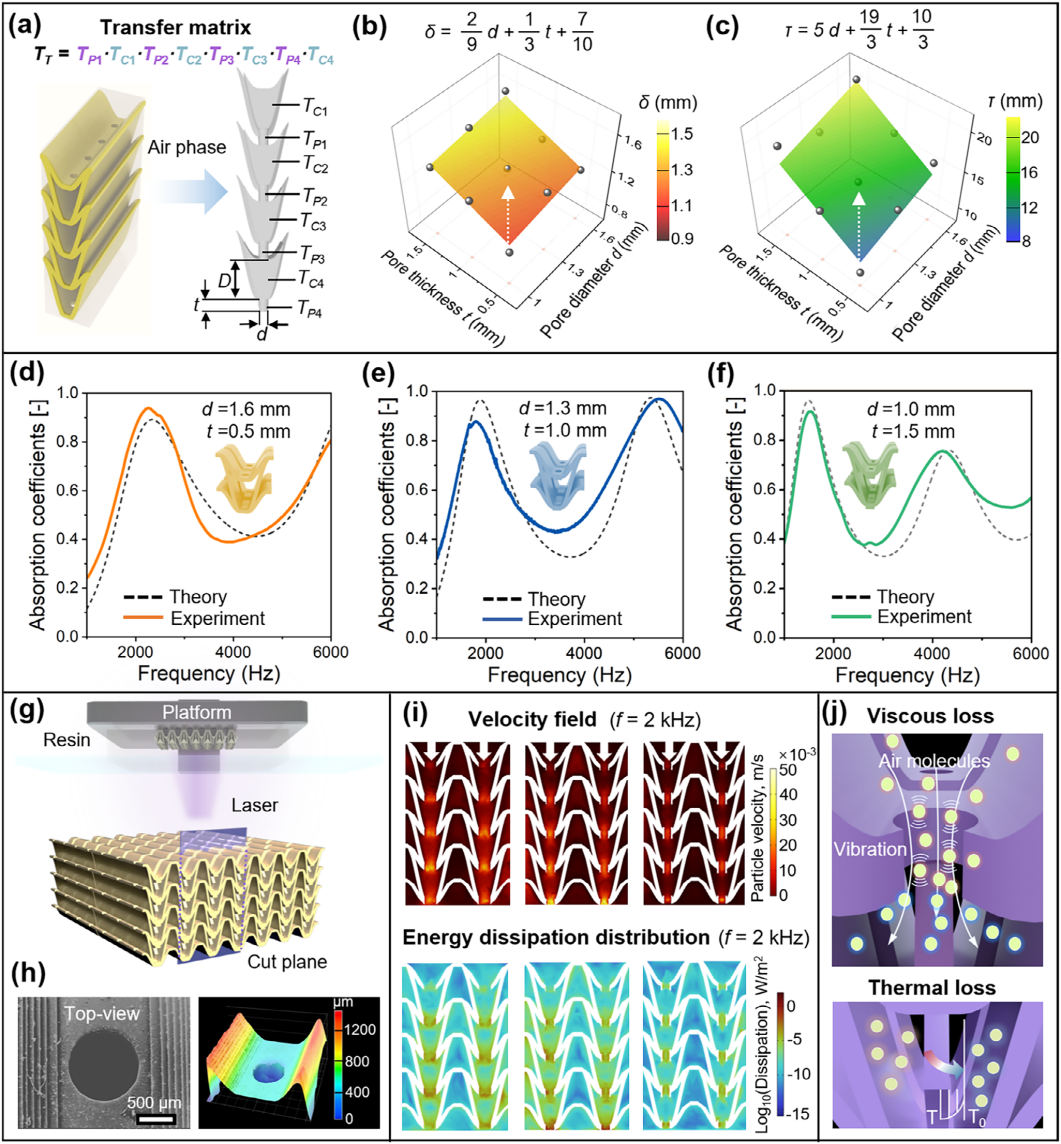
End parameters relevant to the acoustic impedance model and mechanisms of sound absorption: a) Schematic of the transfer matrix method for deriving acoustic impedance. Calibration of b) *δ* and c) *τ* based on geometric parameters. Theoretical and experimental absorption coefficients for d) the sample with *d* = 1.6 mm and *t* = 0.5 mm, e) the sample with *d* = 1.3 mm and *t* = 1.0 mm, and f) the sample with *d* = 1.0 mm and *t* = 1.5 mm. g) Schematic diagram of the fabricated samples using DLP 3D printing technology, h) SEM and super‐depth microscope images of the local pore structures in the fabricated samples. i) Finite element simulation illustrating thermo‑viscous dissipation. j) Schematic diagram of sound wave attenuation mechanisms, including viscous, thermal, and resonant dissipation.

To quantitatively predict the acoustic performance of multilayer biomimetic sound‐absorbing structures, we developed a theoretical model based on acoustic impedance theory, integrating the transfer matrix method with electroacoustic analogies (**Figure** **2**a). The framework accounts for the combined effects of individual pore resistance and cavity coupling, enabling an accurate correlation between geometric pore parameters and sound‐absorption characteristics. Building on the classical theory of perforated‐panel absorbers, the formulation is extended and adapted to our moth‐wing‐inspired architecture, and incorporates geometry‐specific end corrections to capture its unique acoustic behavior.

The MLHR configuration is characterized by two primary components: the narrow perforations and the underlying cavities, represented by the transfer matrices *T_P_* and *T_C_*, respectively.^[^ ^32^ ^]^ The full matrix expressions, impedance combinations, and absorption‐coefficient evaluation are provided in (Section S3, Supporting Information). The perforation layer is represented through its specific acoustic impedance *Z_P_*:^[^ ^33^ ^]^

(1) $$Z_{p}=\frac{1}{\emptyset }\left(i\omega \rho_{e}t+2\tau R_{s}+i\omega \rho_{0}\delta d\right)$$

where *Φ* is the surface porosity, calculated as *Φ* = п*d* ^2^/2*w* ^2^ for our structure, *ω* is the angular frequency, *ρ*_0_ is the density of air under standard conditions (1.21 kg/m^3^), *R_s_* is the surface resistance associated with the oscillatory viscous airflow along the walls and edges of a perforation. *i* is the imaginary unit, *δ* and *τ* are empirical correction factors used to match the non‐standard multi‐layer Helmholtz resonators.

The end correction term 2*τR_s_* in Equation (1) is used to represent the air friction from oscillatory viscous flow at the perforated‐plate surface. This term accounts for the additional resistance to airflow due to the interaction between the air and the surface of the perforation. The resistance end correction factor *τ* accounts for the adjustment in airflow resistance as sound waves propagate through narrow apertures. In addition, the term of i*ωρ*_0_ *δd* in Equation (1) accounts for the added air mass at both ends of the hole. The reactance end correction factor *δ* compensates for the extra vibration depth that extends outward from the perforation. As sound propagates through the pore, the vibrating air column stretches beyond the inlet and outlet, modulating the effective resonance characteristics and contributing to the total acoustic impedance. With increasing *d*, the ratio of the viscous boundary layer to the total cross‐sectional area decreases, allowing smoother mass transfer and thus enhancing the added mass effect. Consequently, the value of *δ* increases with larger pores, reflecting more pronounced reactance contributions.

The end correction terms are expected to follow the dissipative trends observed in physical experiments and are inherently non‐trivial functions of geometrical parameters, including *d*, *t*, and other microstructural features. To accurately capture these dependencies, the empirical correction models were developed by fitting the theoretical absorption curves to the experimental results obtained from homogeneous absorbers (Figure 2b,c). The optimal end correction expressions, expressed in millimeters, are as follows:

(2) $$\tau =5d+\frac{19}{3}t+\frac{10}{3}$$

For biomimetic metamaterials featuring complex internal cavity geometries, a pore‐size‐dependent empirical model for the added mass correction *δ* was similarly derived:

(3) $$\delta =\frac{2}{9}d+\frac{1}{3}t+\frac{7}{10}$$

It is noted that the above end correction expressions were calibrated for the structural parameter range investigated in this work, specifically for pore diameters *d* = 1.0–1.6 mm and plate thicknesses *t* = 0.5–1.5 mm. Within this design space, the empirical end‐correction terms provide accurate predictions, whereas geometries far outside these ranges may require updated *τ* and *δ* obtained through the same fitting procedure. To further validate the accuracy of the proposed biomimetic moth‐wing‐inspired sound absorption model, we conducted both theoretical predictions and experimental measurements on homogeneous absorbers with varying *d* and *t*. Specifically, three geometric configurations were examined: (i) *d* = 1.0 mm, *t* = 1.5 mm; (ii) *d* = 1.3 mm, *t* = 1.0 mm; and (iii) *d* = 1.6 mm, *t* = 0.5 mm. As shown in Figure 2d–f, the predicted absorption spectra exhibit excellent agreement with the experimentally measured curves across the relevant frequency ranges. This strong correlation confirms the predictive capability and robustness of the acoustic impedance model, thereby validating its effectiveness in capturing the sound absorption behavior of structured, pore‐dependent metamaterials.

The precise fabrication of pore structures is essential for obtaining reliable acoustic measurements. As shown in the digital light processing (DLP) setup (Figure 2g), all samples were fabricated layer‐by‐layer with sub‐micron positioning accuracy, ensuring high structural fidelity. The SEM image (Figure 2h) confirms that the printed surfaces are free of cracks and exhibit sharp, burr‐free pore edges, effectively minimizing the impact of geometric imperfections on experimental outcomes. The dimensional deviation of the pore diameter *d* was within ±0.02 mm, and the overall variation in porosity remained within ±0.1% (detailed in Supporting Information). Furthermore, 3D surface reconstruction using a super‐depth microscope reveals excellent agreement between the printed geometries and the theoretical design, with pore dimensions conforming closely to the intended specifications.

Leveraging this high‐precision platform, the study elucidates a synergistic dissipation mechanism within the porous structure: viscous flow resistance and thermal boundary layer losses—under resonant acoustic excitation (Figure 2j). At resonance frequencies, thermoviscous effects dominate due to strong thermal and viscous interactions within the microperforated architecture. When incident sound waves propagate into the pores, the oscillatory motion of air molecules could induce both shear‐induced viscous damping and heat exchange with the pore walls, leading to energy dissipation. To quantitatively capture these effects, a thermoviscous finite element model was constructed in COMSOL Multiphysics (Figure S5, Supporting Information), solving the linearized Navier–Stokes equations (detailed in Section S4, Supporting Information). The numerical simulations successfully capture the thermoviscous effects in a lossless isentropic system. The results indicate that at resonance frequencies, significant viscous and thermal dissipation simultaneously occur within the pore structures.

Figure 2i presents the acoustic particle‐velocity field and spatial distribution of energy dissipation within the pore structures. The incident acoustic waves induce intense molecular oscillations in the confined geometries, and analysis of the tangential velocity field reveals significantly elevated velocity amplitudes near the pore entrances, which decay exponentially along the depth of the pore as the wall frictional effect increases with increasing *t*. Based on the established viscous dissipation energy density model, the viscous power dissipation *W_V_* can be expressed as follows:^[^ ^34^ ^]^

(4) $$W_{V}=\frac{1}{2}\mu \frac{{v_{t}}^{2}}{\emptyset_{V}}$$

where *µ* is the dynamic viscosity of air, *v_t_* is the tangential velocity, and ∅*_V_* represents the characteristic geometric parameter of the viscous boundary layer. This expression highlights the direct dependence of energy dissipation on the local tangential flow velocity and the geometric confinement of the viscous layer.

The thermoviscous simulations further indicate that total dissipation is highly localized within the pores, confirming the dominant role of pore‐resolved mechanisms in acoustic energy attenuation. At an excitation frequency of 2000 Hz, the configuration with *d* of 1.3 mm and *t* of 1.0 mm demonstrates the most pronounced tangential velocity field and corresponding viscous energy dissipation. This optimal response is attributed to the effective resonant coupling between the pore geometry and the incident acoustic wavelength, which generates steep sound pressure gradients and facilitates efficient conversion of acoustic energy into heat. The dissipation trends predicted by the three theoretical models are consistent with the experimentally measured sound absorption coefficients, thereby validating the fidelity of the analytical approaches. Moreover, the study shows that the pore diameter increase enhances the penetration of acoustic waves into the resonator cavity, thus raising the effective acoustic mass and altering the system's impedance characteristics. This modification results in a rightward shift of the structural resonance frequency, offering a means to tune the sound absorption performance across desired frequency bands through geometric design.

### 3.2 Heterogeneous Broadband Absorber

**Figure 3:**
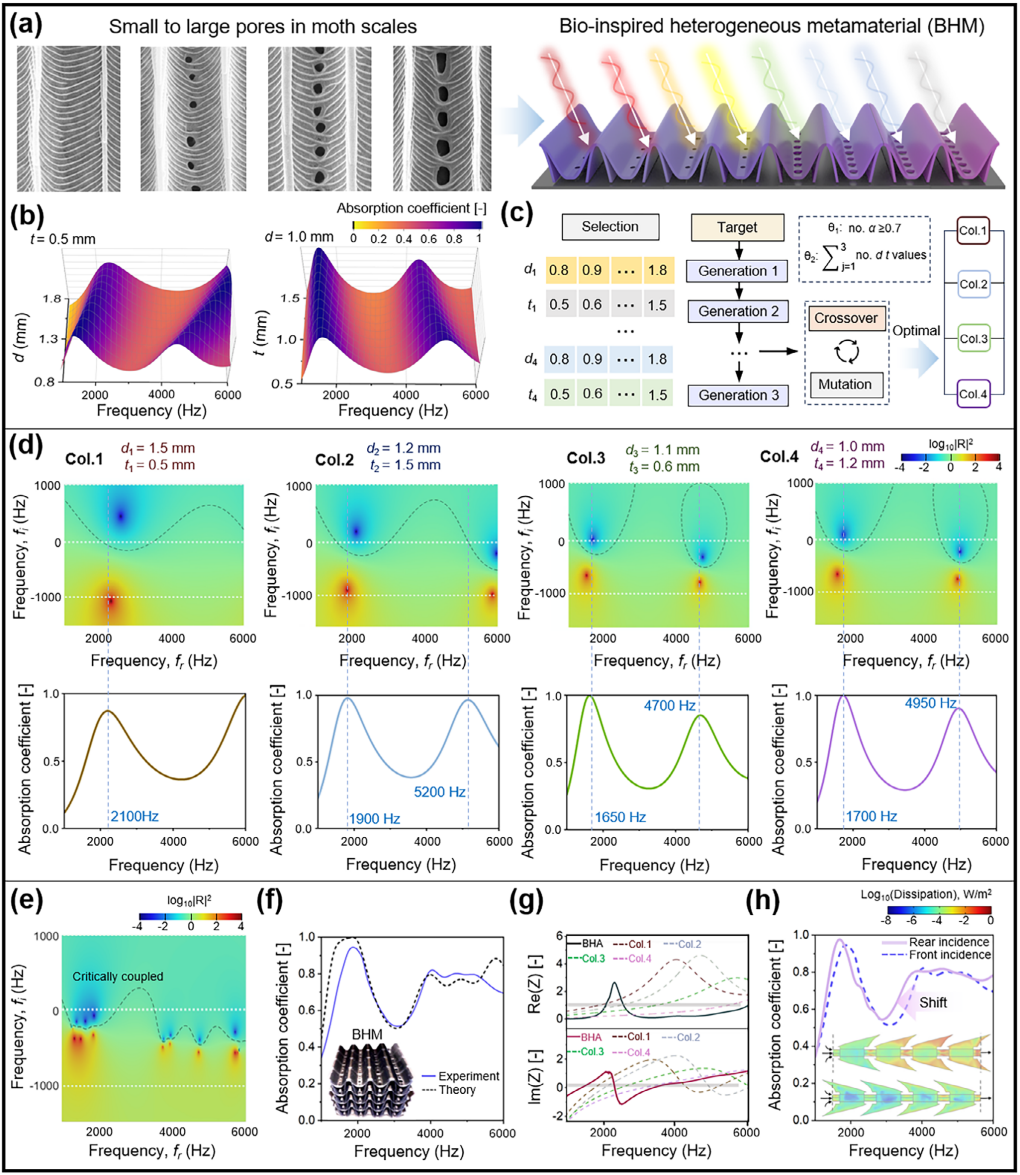
Heterogeneous Helmholtz resonator‐based metamaterial absorber for broadband sound absorption. a) Schematic illustrating the gradient pore architecture inspired by moth wing scales, designed to broaden the acoustic absorption band. b) Frequency responses demonstrating the shift in absorption peaks concomitant with variations in pore diameter and depth. c) Parameter sweep domain and optimization workflow employed within the genetic algorithm framework. d) Complex planes and absorption coefficient curves (*α*) for each of the four constituent resonator columns within the BHM. e) Complex plane for the full BHM, highlighting the presence of conjugate pole–zero pairs indicative of critical acoustic coupling. f) Broadband absorption performance: comparison between experimental measurements and theoretical predictions. g) Real and imaginary components Re(*Z_r_*) and Im(*Z_r_*) of the acoustic impedance of the BHM. h) Improved absorption performance under different sound incidence conditions, highlighting enhanced thermal boundary layer dissipation.

The conventional homogeneous Helmholtz resonators are highly effective at absorbing sound at their resonance frequency but face a fundamental limitation in achieving broadband acoustic absorption, a persistent challenge for architected lattice structures. Drawing inspiration from our earlier observations of moth wing microstructures, we noted that the pores distributed across the scales are spatially heterogeneous in both size and density. As illustrated in **Figure** **3**a, the pore diameter tends to increase as the scales become narrower, while some wider scales lack perforations entirely, suggesting a mechanical rather than acoustic function. This observation implies that spatially graded Helmholtz‐type resonators, mimicking the morphological gradients found in moth wings, can enable multiple, overlapping resonance modes, thereby broadening the absorption bandwidth and enhancing overall acoustic performance. This bioinspired strategy offers a promising avenue to transcend the intrinsic limitations of uniform resonator‐based metamaterials.

To quantify the influence of pore geometry on absorption behavior, we systematically investigated the acoustic performance of resonators with varying *d* and *t*, incremented by 0.1 mm. As shown in Figure 3b, the absorption spectrum is more sensitive to variations in pore diameter than depth. Specifically, increasing *d* shifts the resonant peak toward higher frequencies, while decreasing *t* causes a downward frequency shift. This trend aligns with predictions from the Helmholtz resonance model, where the acoustic mass and cavity stiffness jointly determine the resonant frequency.

However, a single MLHR geometry exhibits only a limited number of resonance peaks. Building on this understanding, we designed a broadband absorber comprising four heterogeneously‐structured unit cells with varying pore geometries arranged parallel to one another, and normal to sound incidence. The broadband absorption behavior arises from the concurrent excitation of multiple resonance modes that operate in tandem, with their overlapping resonance peaks collectively broadening the effective absorption bandwidth. To emulate the graded acoustic response of moth wing scales, a genetic algorithm was employed to optimize the spatial configuration of the unit cells and maximize broadband sound absorption (Figure 3c). Within the design constraints, two independent variables (*d* and *t*) are defined. *D* is coupled with t by the constraint relation of *D* + *t* = 4.5 mm. For each (*d*, *t*) combination, the acoustic impedance *Z_r_*_,_ *_i_* of each column unit was computed using the transfer matrix approach, based on the theoretical framework established in the previous section. The total acoustic impedance *Z_r_*_,_ *_T_* of the four‐unit composite was then determined by applying the parallel impedance summation rule:

(5) $$Z_{r,T}=\frac{4}{\sum_{i=1}^{4}\frac{1}{Z_{r,i}}}$$

Subsequently, the absorption coefficient (*α*) curve for each configuration was calculated over the frequency range of 1000–6000 Hz. The optimization criterion set in the genetic algorithm was to maximize the total number of discrete frequency points, sampled at intervals of 1 Hz, across the said frequency range, for *α* ≥ 0.7. Thus, a greater number of high absorption peaks corresponds to a broader overall absorption bandwidth.

## 4 Results and Discussion

### 4.1 Broadband Sound Absorption

From the genetic algorithm, the optimal configuration yielded pore diameters of *d* = 1.0, 1.1, 1.2, and 1.5 mm, with their respective pore depths of *t* = 1.2, 0.6, 1.5, and 0.5 mm. Here, we term the optimized structure as the bionic heterogeneous acoustic metamaterial (BHM). The absorption mechanism can be interpreted through the relationship between the reflection coefficient |*R*|^2^, resonant modes, and the principle of critical coupling. As illustrated in Figure 3d, the color map represents log_10_|*R*|^2^ as a function of both the real (*f_r_*) and imaginary (*f_i_*) frequency components, revealing the positions of conjugate poles (red) and zeros (blue). The conjugate zeros correspond to frequencies of maximum sound absorption, while the poles denote resonance modes that influence the system's energy dissipation characteristics. The critically coupled states occur when the conjugate zeros align with the real frequency axis (*f_i_* = 0), ensuring maximum acoustic energy dissipation. In contrast, the overdamped systems exhibit zeros below the real axis, while underdamped systems exhibit zeros above it.^[^ ^35^ ^]^ The proximity of these features to the real axis and their alignment with resonance peaks highlight the dissipation characteristics of the structure. The dashed regions in the diagram outline the frequency intervals where *α* ≥ 0.7, corresponding to the ellipsoidal regions surrounding conjugate zeros. The intersections of these regions with the real axis indicate the frequencies of effective absorption in the measured spectrum. Figure 3d also presents the individual absorption spectra of each component. These curves exhibit distinct localized resonance peaks, characteristic of homogeneous resonators, with absorption maxima that align well with their respective conjugate zeros. This confirms the dominance of localized, frequency‐selective resonance behaviors in each unit cell and underscores the advantage of assembling heterogeneously tuned components to achieve broadband performance.

As illustrated in Figure 3e, the BHM exhibits a distribution of seven pairs of conjugate poles and zeros across the entire frequency range. Notably, the resonance peak near 2000 Hz aligns with a critical coupling state, while other resonant peaks demonstrate mildly overdamped behavior. Each individual unit column contributes only one to two pairs of conjugate poles and zeros; however, their aggregate precisely matches the total number observed in the complete BHM. This observation implies that the overall acoustic response of the BHM can be interpreted as a linear superposition of the resonance characteristics of its constituent units. Further analysis reveals that within the efficient sound absorption range (*α* ≥ 0.7), the BHM generates a continuous broadband absorption band through the synergistic interaction of adjacent conjugate zeros. This cooperative behavior significantly broadens the effective absorption range compared to conventional single‐resonant unit structures. Indeed, the sound absorption curves of the BHM, as shown in Figure 3f, reveal high absorption along with a broad effective bandwidth. The measured results exhibit a smooth and elevated absorption profile across the broadband range of 1000–6000 Hz, with an average absorption coefficient of 0.714. Notably, 63% of the frequency points within this range exceed the threshold of *α* = 0.7, underscoring the effectiveness of the design. Meanwhile, the excellent agreement between theoretical and experimental curves confirms the high fidelity of our analytical acoustic model. Some minor discrepancies can be attributed to the inherent characteristics of the layer‐by‐layer additive manufacturing process, which introduces surface roughness and discrete interfacial discontinuities between printed layers. Such imperfections deviate from the assumptions of idealized modeling, thereby contributing to the observed variances between theoretical and experimental absorption performance. Figures S6 and S7 (Supporting Information) provide crucial finite element simulation insights into the micro‐scale physical mechanisms underpinning the broadband sound absorption observed in the BHM. Figure S6 (Supporting Information) illustrates that when sound waves encounter the BHM, the particle velocity significantly increases, channeling acoustic energy into vibrational modes, with high‐velocity regions indicating localized resonance peaks at various frequencies. Figure S7 (Supporting Information) reveals significant and distributed energy dissipation across a broad range of frequencies, confirming that the heterogeneous design facilitates multiple dissipation pathways, cumulatively resulting in a wide absorption spectrum.

The broadband sound absorption behavior of the heterogeneous modified Helmholtz resonator absorber can be further elucidated by analyzing the relative acoustic impedance of its constituent units, as depicted in Figure 3g. In this context, Re(*Z_r_*) and Im(*Z_r_*) represent the real and imaginary components of the relative surface impedance, respectively. Physically, the real part Re(*Z_r_*) reflects the degree of energy dissipation within the structure, while the imaginary part Im(*Z_r_*) indicates the reactive characteristics, corresponding to the specific frequencies at which dissipation occurs. As derived from the theoretical model, the ideal condition for maximum sound absorption (*α* = 1) is achieved when Re(*Z_r_*) = 1 and Im(*Z_r_*) = 0. Under this critical coupling condition, the surface impedance of the structure matches that of air, enabling complete acoustic energy transfer and minimal reflection. The plotted curves of Re(*Z_r_*) = 1 and Im(*Z_r_*) therefore, illustrate the degree of impedance matching across the frequency spectrum.^[^ ^36^ ^]^ The heterogeneous architecture introduces multiple resonant modes, in which each mode could be tailored to different frequency bands. These modes work in concert to enable optimal impedance matching over a broad frequency range. At any given frequency, the specific unit cells can dominate the impedance characteristics, ensuring localized energy dissipation and maintaining high absorption performance. Compared to homogeneous resonators, the composite structure exhibits significantly closer alignment of Re(*Z_r_*) and Im(*Z_r_*) to their ideal values across the operating bandwidth. This confirms the efficacy of the heterogeneous design in overcoming the narrowband limitations of conventional absorbers and achieving efficient broadband sound absorption.

Figure 3h presents the absorption coefficients of the BHM under two different sound incidence configurations. Remarkably, altering the direction of sound incidence leads to a notable enhancement in overall acoustic performance. In the initial configuration, the BHM exhibited an average absorption coefficient of 0.714, with 63.0% of frequency points within the 1000–6000 Hz range achieving absorption values greater than or equal to 0.7. Upon reversing the incidence surface, the average absorption coefficient increased to 0.742, with 72.0% of the frequency points exceeding the 0.7 threshold. This performance is highly competitive, as shown in Table S1 (Supporting Information). It surpasses most microlattice resonators and microperforated panels with comparable thickness and frequency range and rivals the broadband absorption of certain aerogels. This is really a highly challenging result for conventional Helmholtz resonators that often require greater thickness or complex multilayer designs to achieve similar broadband efficiency.

This directional dependence highlights the asymmetric acoustic response of the heterogeneous architecture, likely stemming from spatial variations in pore geometry and cavity distribution, which promotes more effective resonance coupling and impedance matching under specific excitation directions. For conventional multi‐layer Helmholtz resonators, sound absorption primarily arises from viscous losses due to airflow through the resonator apertures at resonance. Notably, viscous dissipation remains largely unaffected by changes in the direction of sound incidence. This indicates that the observed enhancement in absorption performance is primarily driven by increased thermal boundary layer dissipation. Altering the incidence surface introduces more complex internal reflection paths, which modify the standing wave distributions and effectively lower the system's resonant frequencies. The simulation results further reveal that, for a given aperture size, changing the incidence geometry causes thermal dissipation to concentrate at geometrically confined corners—enhancing local heat loss by two orders of magnitude compared to standard micro‐perforated panels (Figure S8, Supporting Information). This intensification arises from repeated sound wave reflections in confined spaces, where air molecules undergo continuous compression and rarefaction, leading to intensified thermal losses. These findings underscore the critical role of structural geometry, particularly thermal boundary effects, in governing and optimizing broadband sound absorption performance.

### 4.2 Application of Helmet for Noise Control

**Figure 4:**
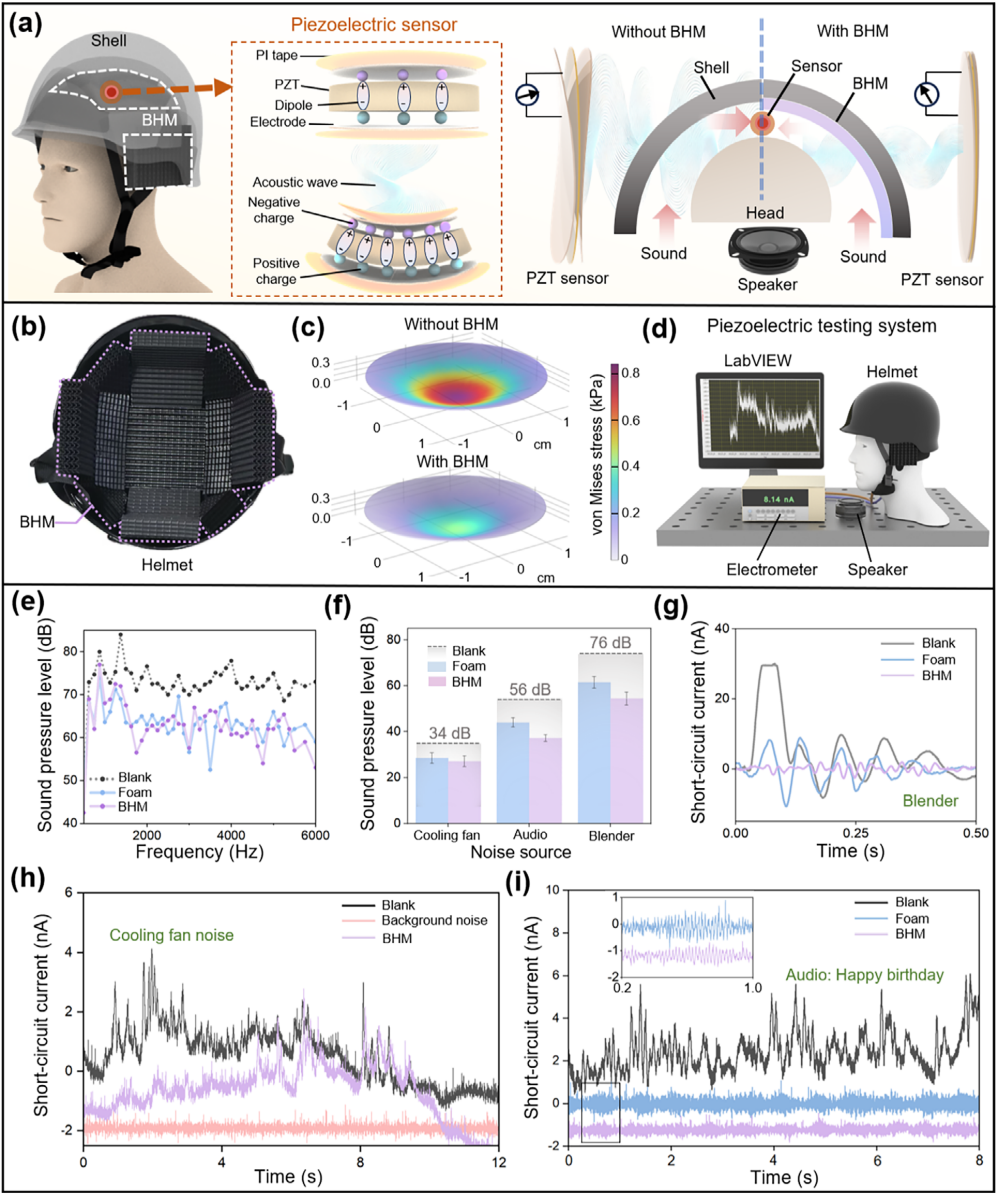
Integration of BHM into a helmet for enhanced noise reduction. a) Schematic illustration of the working principle of a piezoelectric (PZT) sensor embedded in the helmet interior, transducing incident acoustic waves into electrical signals. b) Interior photograph of the helmet, highlighting the BHM‐based acoustic absorption liners. c) Finite element simulation of stress distribution across the helmet shell under identical acoustic excitation, demonstrating significant stress mitigation after introducing BHM. d) Schematic illustration of the experimental setup for acoustic performance testing. e) Measured sound pressure level (SPL) spectra across 1000–6000 Hz for three configurations: bare helmet, helmet with conventional foam, and helmet with BHM. f) Bar graph comparing the average SPL reduction under three representative noise sources for the same configurations (mean ± SD, *n* = 3). g) Short‐circuit current responses from the PZT sensor under high‐intensity noise from a high‐speed blender. h) Electromechanical response of the PZT under continuous low‐level noise from a cooling fan. i) Short‐circuit current response during musical input (“Happy birthday”); inset: zoomed‐in view of the low‐amplitude region, highlighting reduced electrical output due to BHM‐induced noise attenuation.

The BHM developed in this study presents a promising solution for real‐world noise mitigation. Its multifunctional design renders it suitable for noise‐sensitive scenarios, including personal protective equipment, high‐speed rail interiors, and drone noise management (Figure S9, Supporting Information). To further validate its applicability in practical systems, we integrated the BHM into a helmet as a representative noise‐control application. During helmet wearing, the narrow air gap between the wearer's ear and the helmet shell serves as a primary acoustic transmission path, making it a critical zone for effective noise attenuation. As shown in the schematic diagram (**Figure** **4**a), the BHM liners are attached to the inner surface of the helmet, serving as the functional padding layer (Figure 4b), while a piezoelectric sensor is embedded to harvest residual acoustic energy. The sensor is positioned at the top of the helmet, which represents the region of strongest global acoustic reflection and vibration coupling within the shell rather than a purely local ear position. This configuration enables a consistent evaluation of how effectively the BHM liner suppresses both shell‐transmitted and inner‐reflected sound energy within the confined air cavity. The schematic on the right contrasts the direct transmission path in an unmodified helmet (left) with the attenuated acoustic pathway in the BHM‐lined helmet. As shown in Figure 4c, piezoelectric coupling simulations performed using COMSOL reveal the regulatory effect of the BHM on the piezoelectric response of embedded sensors. The setup for these simulations is detailed in Section S6 (Supporting Information). Under exposure to a free acoustic field, the sensor experiences substantial mechanical deformation due to the acoustoelastic effect, leading to pronounced stress concentration at its central region (Figure S10, Supporting Information). This localized stress distribution induces the generation of polarized charges, resulting in a peak electric field magnitude of 15.4 V m^−1^ (Figure S11, Supporting Information). Upon integration of the BHM, the effective sound pressure acting on the sensor's surface is significantly attenuated through porous viscoelastic dissipation. The internal viscous losses within the microstructured pores of the BHM effectively dissipate incident sound energy, demonstrating its potential for wearable acoustic protection systems. To quantitatively assess the system's performance, we constructed a mannequin‐mounted experimental test bench (Figure 4d), comprising a calibrated loudspeaker operating over the 1000–6000 Hz frequency range, a Brüel‐type microphone for monitoring the incident sound pressure level (SPL), a PZT sensor connected to a Keithley electrometer with 10 pA resolution (the acoustic frequency response of the PZT sensor is shown in Figure S12, Supporting Information), and a LabVIEW‐based data acquisition system. Within this setup, the helmet shell functions as a rigid acoustic backing, while the BHM liner serves as the dissipative medium. The inclusion of the BHM liner leads to a measurable reduction in internal sound levels, primarily due to the energy dissipation occurring within its architected microstructure. Therefore, the observed attenuation can be considered a proxy for evaluating the BHM's in situ sound absorption performance under realistic operational conditions. Figure 4e compares the steady‐state SPL spectra for three liner configurations. BHM outperforms bare foam across 1000–6000 Hz, lowering SPL by an average of 10.2 dB relative to the blank helmet, versus 9.7 dB for foam. The most pronounced edge (≈3 dB) occurs in the crucial 1500–2000 Hz.

In practical noise reduction tests, the BHM achieved noise reduction of 6 dB for cooling fan noise (low‐frequency dominated), 19 dB for a song (“Happy birthday” audio), and 17 dB for high‐speed blender noise, outperforming the commercial foam in all cases (Figure 4f). Notably, the BHM‑lined helmet exhibits the largest suppression under the high‐speed blender noise. In Figure 4g, the blank helmet configuration registers a short‑circuit current approaching 30 nA, while the introduction of a conventional foam liner suppresses the peak by only 50 %. In stark contrast, the current trace recorded with the BHM liner is effectively compressed to 4 nA, confirming that the architected microstructure dissipates broadband mechanical energy far more effectively. A similar trend is observed for low‐frequency cooling‑fan excitation (Figure 4h): the blank configuration exhibits transient bursts exceeding 4 nA, whereas the BHM configuration yields a notably reduced current response that approaches, but remains distinguishable from the electrical baseline. During mid‑frequency audio playback of “Happy Birthday” (Figure 4i), the BHM again outperforms foam, producing a markedly smoother and lower current trace that mirrors the SPL reductions summarised in Figure 4f. The additional comparisons of acoustic current responses under human speech further confirm the superior noise attenuation performance of the BHM over conventional foam (Figure S13, Supporting Information).

Together, these electromechanical signatures substantiate the broadband sound‑absorption capability of the BHM's architected, porous microstructure and highlight its utility for advanced in situ noise protection. In addition, the integrated helmet design demonstrates clear advantages over traditional earplugs, which seal the ear canal and often cause discomfort during long wear while offering limited adaptability. It exhibits frequency‐selective absorption with strong attenuation in the 1000–6000 Hz range, corresponding to the dominant noise spectrum of engineering equipment. In contrast, most earplugs uniformly attenuate sound across frequencies, reducing speech perception and environmental awareness. The helmet design provides an ergonomic all‐in‐one alternative for moderate‐noise environments.

### 4.3 BHM‐Lined Helmet with Mechanical and Thermal Resistance

**Figure 5:**
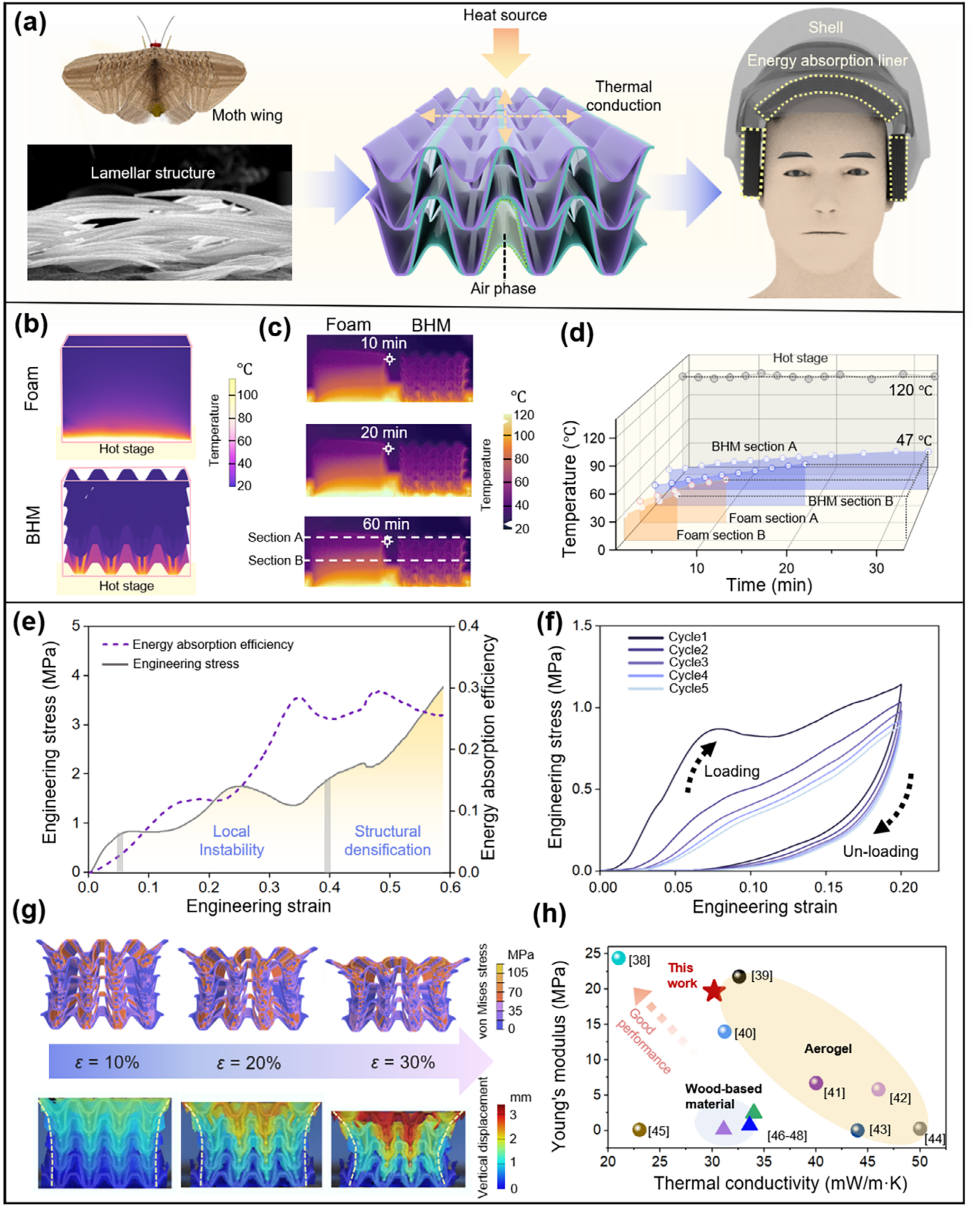
Bioinspired multifunctional BHM integrating mechanical energy absorption and thermal insulation for helmet liner applications. a) Conceptual illustration of the lamellar structure of moth wings, serving as the design basis for the energy‐absorbing liner integrated into the helmet shell. b) COMSOL‐simulated temperature distribution when the hot stage is held at 120 °C. c) Infrared thermographs comparing commercial foam and BHM at 10, 20, and 60 min of exposure on a heated stage; dashed lines A and B indicate regions used for temperature profiling. d) Time–temperature profiles along lines A and B, with a reference threshold (47 °C) marked by the dotted horizontal line. e) Quasi‐static compression response of the BHM, illustrating the progression from local buckling to structural densification, along with the corresponding energy absorption efficiency curve. f) Five‐cycle loading–unloading test indicating clear hysteresis behavior and robust mechanical recoverability. g) Comparison between ABAQUS finite element predictions (top row) and DIC‐measured vertical displacement fields (bottom row) at engineering strains of *ɛ* = 10%, 20%, and 30%. h) Ashby plot benchmarking the BHM's effective thermal conductivity versus Young's modulus, positioning it among advanced aerogels and wood‐derived materials in terms of multifunctional performance.

Having established the BHM as an effective broadband acoustic absorber under complex and real‐world sound fields, we next investigated whether a single liner could simultaneously meet the stringent mechanical and thermal performance requirements of protective helmets. This challenge led us back to the moth wing—an archetype of multifunctionality in nature. As illustrated in **Figure** **5**a, moth wings feature alternating chitin lamellae and air cavities: the trapped air (thermal conductivity ≈26 mW·m^−1^·K^−1^) disrupts conductive heat flow, the quasi‐photonic lamellae reflect incident thermal radiation, and the staggered layering dissipates impact energy scale‐by‐scale. Inspired by this natural design, we translated the motif into a periodic lattice structure and embedded it between the helmet shell and the headform, with the goal of integrating thermal insulation, energy dissipation, and lightweight architecture into a single insert.

To assess thermal insulation performance, we conducted a comparative experiment using both commercial foam and BHM samples placed on a hot stage maintained at 120 °C. The steady‐state temperature distributions predicted by COMSOL simulations (Figure 5b) reveal pronounced differences in thermal behavior under identical boundary conditions (with the bottom “hot stage” serving as the heat source). In the commercial foam, the heat flux follows a predominantly vertical path, indicating direct and rapid thermal conduction. In contrast, the BHM displays a more tortuous, layered heat flow pattern, demonstrating that its internal microarchitecture elongates the conduction path and effectively impedes heat transfer.

The infrared thermographs captured at 10, 20, and 60 min on the hot stage (Figure 5c) vividly validate the simulation predictions. The temperature profiles extracted along section A (surface) and section B (mid‐thickness) are plotted in Figure 5d, confirming that the same bioinspired geometry responsible for mechanical deformation also introduces a tortuous thermal transport pathway. A representative “polymer lamella–air cavity” unit and the corresponding COMSOL‐calculated temperature field under a 120 °C boundary condition are shown in Figure 5b. In commercial foam, the heat flux follows a predominantly vertical path, facilitating rapid thermal conduction. In contrast, BHM forces the heat to traverse an elongated, zigzag pathway, with the air cavities serving as the dominant contributor to overall thermal resistance, thereby significantly reducing the composite's effective thermal conductivity.^[^ ^37^ ^]^ As a result, the surface temperature of the BHM stabilizes at 47 °C, 26 °C lower than that of the commercial foam, and requires 23 min to reach this equilibrium, more than twice the time required by the foam (11 min). A theoretical derivation (see Supporting Information for details) further corroborates the superior thermal insulation performance of the BHM, yielding an effective thermal conductivity of 30.2 mW·m^−1^·K^−1^.

The mechanical characterization confirms that the BHM exhibits an auxetic behavior under large deformation. The mechanical response and energy absorption performance of BHM were first evaluated under uniaxial compression. As shown in Figure 5e, the engineering stress‐strain curve exhibits typical three‐stage deformation characteristics, closely tied to its cellular lattice architecture. At small strains (ɛ < 0.05), the response is linear, indicating that deformation is primarily governed by the elastic bending of concave structural elements. The energy absorption efficiency at this stage remains low. As strain increases, a serrated plateau emerges in the stress–strain curve, corresponding to localized buckling and layer‐wise collapse within the lattice. These events produce intermittent stress drops followed by partial recovery, as the successive layers are progressively engaged. During this phase, the energy absorption efficiency rises markedly, increasing from 0.02 to 0.28. When *ɛ* exceeds 0.4, the structural elements begin to fail more extensively, leading to a sharp increase in stress. This is accompanied by pronounced lateral contraction due to the structure's negative Poisson's ratio behavior, resulting in densification and mechanical reinforcement. The compressive load required for further deformation increases substantially, and the energy absorption efficiency reaches a maximum at *ɛ* = 0.47. Finite element analysis of the dynamic crushing process validates the deformation mechanism of BHM and confirms its consistency with experimental observations (Figure S14, Supporting Information).

Figure 5f presents the mechanical response of BHM under five loading–unloading cycles at 20% strain. During the loading process of the first cycle, both the structural stiffness and stress reached a high level, while the unloading path significantly deviates from the loading path, indicating a decrease in stiffness. This behavior is attributed to the buckling of concave structural units and localized plastic deformation at node junctions. Notably, the negative Poisson's ratio effect induces multidirectional contraction during compression, which promotes the tight interlocking between adjacent lattice units. This interlocking effectively suppresses crack propagation and preserves the overall structural integrity. In the subsequent cycles, the peak stress amplitude gradually diminishes, yet the area enclosed by the hysteresis loop stabilizes. This suggests that, following initial damage, BHM transitions into a new dynamic load‐bearing regime. The partial recovery of local buckling upon unloading is facilitated by the auxetic effect, while unbuckled elements realign due to structural densification, optimizing load distribution. This behavior ensures stable and repeatable energy dissipation, making BHM a strong candidate for applications in vibration damping and impact mitigation.

To further elucidate the underlying deformation mechanisms of BHM, we conducted both digital image correlation (DIC) experiments (Figure S15, Supporting Information) and finite element simulations using Abaqus/Explicit (Figure 5g). Figures 5g and S16 (Supporting Information) illustrate the displacement field distribution at three different strain levels (*ɛ* = 10%, 20%, and 30%). At *ɛ* = 10%, both the U‐displacement and V‐displacement fields exhibit smooth and symmetric distributions, indicating that deformation is primarily elastic and uniform at this stage. As strain increases to 20%, the localized high‐displacement regions emerge in the V‐displacement field, signifying the onset of local buckling within BHM. Simultaneously, the U‐displacement field reveals a lateral displacement pattern, suggesting that the negative Poisson's ratio effect induces the lateral reorganization of the structure during compression. The displacement distribution gradually extends laterally, highlighting the synergistic deformation between the primary structure and internal supports. At *ɛ* = 30%, the displacement field exhibits pronounced localized deformation, with sharp gradients between red and blue regions. A significant number of concave structures collapse, leading to overall structural densification. This auxetic behavior enhances energy absorption and structural reinforcement under large strains. The simulation results indicate that the deformation patterns of BHM closely match digital image correlation measurements, accurately reproducing its local buckling modes, lateral contraction characteristics, and densification trends.

The intricate coupling between mechanical deformation and acoustic functionality of the metamaterial was further investigated (detailed in Section S10, Supporting Information). As plastic strain increases, the transition from uniform volume redistribution to the collapse of interlayer struts leads to a progressive reduction in cavity volume (Figure S17, Supporting Information). Consequently, the sound absorption spectrum exhibits a distinct rightward shift of the main resonance peak and a decrease in peak magnitude (Figure S18, Supporting Information). In addition, the long‐term stability of the BHM was evaluated under cyclic loading and alternating hygrothermal exposure (detailed in Section S11, Supporting Information). After 2000 compression cycles, the BHM showed stress softening and a modest drop in peak absorption with a slight rightward shift of the main resonance, which is consistent with microcrack‐induced increases in effective porosity. The hygrothermal aging reduced the elastic modulus but left band placement essentially unchanged, with a small decrease in low‐frequency amplitude (Figure S19, Supporting Information).

Finally, benchmarking the BHM on an Ashby map (Figure 5h) underscores the multifunctionality achieved by this cross‐scale design. With an effective thermal conductivity of *λ* = 30.2 mW m^−1^ K^−1^ (the calculation process is detailed in Section S9, Supporting Information) and a Young's modulus of 19.7 MPa, the BHM achieves excellent thermal insulation while maintaining high mechanical strength and structural stability. Compared with aerogels and wood‐based foams (Table S2, Supporting Information), the BHM provides a more balanced combination of low thermal conductivity, high energy dissipation capacity, and broadband acoustic absorption.^[^ ^38^, ^39^, ^40^, ^41^, ^42^, ^43^, ^44^, ^45^, ^46^, ^47^, ^48^ ^]^ To better contextualize this advancement, Figure S20 (Supporting Information) is presented to compare the structure, function, and potential application scenarios of the BHM and conventional Helmholtz resonators. The traditional Helmholtz resonators mainly rely on large cavity depth to achieve low‐frequency absorption, but they are usually rigid, thick, and lack multi‐functional integration. In contrast, the BHM with graded pores, structural anisotropy, and ridge‐cross‐rib architecture achieves multifunctionality in a thin and lightweight configuration. In practical applications such as helmet liners, although DLP‐based fabrication yields a higher unit cost than mass‐produced foams, the BHM is suited to scenarios that demand digitally tunable acoustic bands, mechanical robustness/energy dissipation, and thermal insulation, thereby addressing the performance limitations of uniform foams. Consequently, the BHM's property suite matches the key requirements of next‐generation protective and structural equipment.

## 5 Further Discussion

Overall, this study provides a fundamental proof of concept for the design and application of the BHM; however, further research is required to advance its transition toward practical implementation in three main directions: from the manufacturing perspective, DLP‐based 3D printing offers unique advantages in realizing complex microarchitectures but remains limited by fabrication efficiency and cost, which hinder large‐scale deployment. The future work will focus on exploring advanced scalable processes such as microinjection molding, aiming to preserve microscale structural precision while overcoming constraints on component size and production throughput. From the structural design perspective, the current optimization primarily addresses frequency‐domain acoustic responses without explicitly accounting for mechanical coupling effects or manufacturing constraints. Developing a multi‐physics, multi‐objective co‐optimization framework that integrates genetic algorithms with AI‐driven surrogate modeling will be crucial to enhancing both the overall performance and design efficiency of multifunctional metamaterials. From the material system perspective, improving long‐term service reliability remains a key challenge. To enhance fatigue resistance, flexible segments such as polyurethane acrylate will be incorporated to toughen the matrix, complemented by geometry optimization of load‐bearing structures to delay damage evolution. This can also significantly enhance mechanical compliance and adaptability while maintaining the designed geometry, and improve the formability of the metamaterial on complex curved substrates. To mitigate hygrothermal degradation, the hydrophobic modifications using fluorinated or siloxane‐based monomers, or plasma surface treatments, will be employed to suppress moisture‐induced deterioration. Furthermore, to ensure safety under extreme conditions, integration of high‐efficiency flame‐retardant fillers will be essential to achieving the improved fire resistance.

## 6 Conclusion

In conclusion, inspired by the graded pore architecture and layered structure of moth wings, we have developed a novel metamaterial (BHM) that is capable of simultaneous broadband sound absorption, thermal insulation, and mechanical energy dissipation. Leveraging genetic algorithm–driven optimization, we first translated the BHM into a heterogeneous array of Helmholtz resonators with synergistic resonance modes tailored for broadband sound absorption. Our BHM design was realized and validated through DLP 3D printing. Experimental validation revealed a high average sound absorption coefficient of 0.742 across the 1000–6000 Hz frequency band. The structure also exhibits a negative Poisson's ratio under large deformation and maintains stable energy absorption performance under cyclic compressive loading, reaching an energy absorption efficiency of 0.28 and demonstrating reliable auxetic behavior. The thermal measurements reveal that the metamaterial's alternating polymer–air architecture reduces effective thermal conductivity to 30.2 mW·m^−1^·K^−1^, achieving surface temperatures over 26 °C lower than commercial foams under identical heat flux. When implemented as a helmet liner, the metamaterial achieved practical broadband noise reduction of up to 19 dB and reduced electromechanical transduction by >80%, confirming its in situ efficacy under real‐world acoustic conditions. These results demonstrate a novel materials design framework in which biological inspiration and structural optimization converge to overcome conventional performance trade‐offs, opening new avenues for multifunctional protective systems in aerospace, transportation, personal safety, and other demanding engineering environments.

## 7 Experimental Section

### Fabrication

The design of the bionic heterogeneous acoustic metamaterial (BHM) was created using SolidWorks for computer‐aided design (CAD), and the corresponding models were exported as STL files for 3D printing. All manufacturing was carried out using the Asiga Max X27 (Asiga, Australia) digital light processing (DLP) 3D printer. The filling material used for 3D printing was Anycubic's Tough Resin Ultra, a UV‐curable acrylate‐based photopolymer resin composed mainly of methacrylate monomers. The optimized printing parameters were as follows: layer thickness of 100 µm, light intensity of 5 mW·cm^−2^, and layer curing time of 3 s. The printer's native XY pixel pitch of 27 µm permitted faithful reproduction of the narrow concave struts, while the build platform was pre‐heated to 30 °C to reduce resin viscosity and ensure uniform layer spreading. After printing, the samples were thoroughly washed with isopropyl alcohol for 15 min and post‐cured in the Asiga Flash UV curing chamber for a total duration of 1 h. Following these steps, sound absorption and mechanical property tests were conducted.

### Materials and Characterization

The moth carcasses used in this study were collected from the western Sichuan Basin, China. Collected specimens were stored in desiccators to prevent moisture‐induced distortion of the delicate wing scales before imaging. The 3D‐printed samples were also observed locally using a super‐depth‐of‐field 3D microscope (VHX‐1000C, Keyence, Japan). The thermal response and heat management properties of the samples were assessed using an infrared thermal imaging camera (Testo 872‐2, Testo SE & Co., Germany). Quasi‐static compression tests were conducted on the samples using a Shimadzu universal testing machine, with a strain rate of 0.001 s^−1^, following the ISO 13 314 standard. Deformation of the samples was captured with a digital camera. The fatigue tests were conducted on a universal testing machine under a strain rate of 0.01 s^−1^ and a strain amplitude of 10 %, with the samples subjected to 2000 compression cycles to evaluate cyclic durability and acoustic stability. The initial load‐displacement data from the experiments were converted into engineering stress‐strain data for further analysis. Sound absorption tests were performed using a dual‐microphone impedance tube method in accordance with ISO 10534‐2 standards. Thermal images were captured with a handheld infrared camera (M600, InfiRay), and thermal conductivity of the raw material was measured using a thermal conductivity analyzer (Trident, CTherm) with a modified transient plane source (MTPS) method. The BSWA SW477 series impedance tube (30 mm in diameter) and BSWA Tech's BSWA MPA416 microphone were used for the sound absorption measurements. Data were collected using VA‐Lab acoustic measurement software, with samples positioned snugly within the impedance tube (30 mm diameter) for testing. The sound pressure level (SPL) was monitored using a ½‐inch microphone (Brüel & Kjær Type 4966) coupled to a high‐temperature preamplifier (Type 1706). For the helmet tests, the microphone capsule was mounted on the inner surface of the helmet top, approximately above the geometric center of the head of the manikin, with its diaphragm facing inward toward the cavity to capture the incident acoustic field. The preamplifier body was laid horizontally along the inner shell of the helmet to minimize intrusion into the cavity. The signal cable was lined neatly along the inner wall to avoid the flow‐induced noise or contact vibration. Before the measurement, a field calibration was performed at 1 kHz using a B&K Type 4231 sound calibrator (94 dB). The hygrothermal aging tests (30–60 °C, 85–95 % relative humidity, 24 h per cycle for 10 cycles) were performed using a high–low temperature and humidity test chamber (Nanjing Hilbert Instrument Equipment Co., Ltd., China).

### Statistical Analysis

The absorption coefficient–frequency curves obtained from impedance‐tube measurements were preprocessed in Origin using the Savitzky–Golay smoothing method to reduce random noise and improve signal readability. A window size of 20 and a polynomial order of 2 were applied, with no edge effects correction. The sound pressure level results were analyzed in Origin, and the error bars represent the mean ± standard deviation (*n* = 3).

## Conflict of Interest

The authors declare no conflict of interest.

## Author Contributions

H.P. and W.Z. conceived the conceptualization. H.Y., Y.C., and W.Z. performed formal analysis. X.L., H.Y., Y.C., H.P., T.L., and W.Z. performed methodology. X.L., N.Z., H.Y., X.X.W., and S.D. performed the software. H.P., H.Y., Z.X., Q.L., and W.Z. performed validation. H.P., H.Y., M.Z., X.W., and W.Z. performed the investigation. H.P. performed writing ‐ original draft. H.Y., X.L., Y.C., W.Z. performed writing ‐ review & editing. X.L., Y.C., and W.Z. performed supervision.

## Supporting information



Supporting Information

## Data Availability

The data that support the findings of this study are available from the corresponding author upon reasonable request.
