# Supplementary material for: Moth‐Wing‐Inspired Multifunctional Metamaterials
Source: Adv Mater. 2025 Dec 16;38(9):e15350. doi: 10.1002/adma.202515350 (PMC12902595; doi:10.1002/adma.202515350)
Supplement: Supplementary file 1 — Supporting Information [file ADMA-38-e15350-s001.docx]

Supporting Information

Moth-Wing-Inspired Multifunctional Metamaterials

*Haoran Pei, Hang Yang, Ning Zhang, Tian Li, Xinxin Wang, Miao Zhao, Shuwei Ding, Xin Wang, Qinniu Lv, Zijie Xu, Yinghong Chen*, Xinwei Li*, Wei Zhai**

H. Pei, X. Wang, Q. Lv, Z. Xu, Y. Chen

State Key Laboratory of Advanced Polymer Materials, Polymer Research Institute of Sichuan University, Chengdu, 610065, China

E-mail: [johnchen@scu.edu.cn](mailto:johnchen@scu.edu.cn)

H. Pei, H. Yang, N. Zhang, T. Li, X.X. Wang, S. Ding, W. Zhai

Department of Mechanical Engineering, National University of Singapore, Singapore, 117575, Singapore

E-mail: [mpezwei@nus.edu.sg](mailto:mpezwei@nus.edu.sg)

X. Li

Newcastle University in Singapore, Faculty of Science, Agriculture, and Engineering, Newcastle University, Newcastle upon Tyne NE1 7RU, UK

E-mail: [xinwei.li@newcastle.ac.uk](mailto:xinwei.li@newcastle.ac.uk)

M. Zhao

School of Mechanical and Electrical Engineering, University of Electronic Science and Technology of China, Chengdu, 611731, China

**S1. Performance advantages of bionic heterogeneous acoustic metamaterial (BHM) over conventional foams and lattices**

Compared to traditional foams, the novel BHM demonstrates enhanced stress–strain behavior, characterized by a multistep deformation mechanism that enables higher compressive strength and improved energy dissipation. Meanwhile, relative to conventional acoustic lattices, which typically exhibit narrowband absorption peaks, the BHM achieves significantly improved and more uniform absorption across a broad frequency range. This combination of mechanical reinforcement and broadband sound attenuation effectively addresses the core challenges of traditional liners and highlights the advantages of bioinspired architectural design in multifunctional acoustic materials (Figure S1).


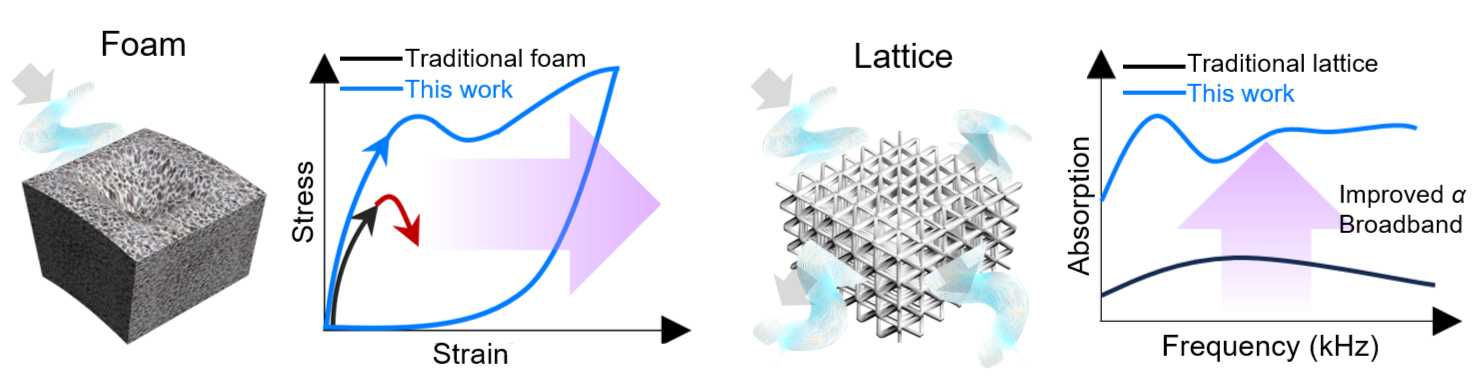


**Figure S1.** Traditional foam and lattice deficiencies in acoustic and mechanical properties.

**S2. The microstructural characterization of moth wing scales and comparative analysis with other insects**

The microstructure of the moth wing scale was observed using a field-emission scanning electron microscope (SEM, Helios G4 UC, USA) with an acceleration voltage of 15 kV and a laser confocal microscope (LCM, Zeiss LSM700, Germany). As shown in Figure S2, the 3D surface topography obtained from LCM reveals pronounced height variations across the overlapping scales, particularly between the scales. The height gradient spans from approximately 5 µm to over 45 µm, and the layered and tile-like arrangement indicates a stacking pattern. These height variations may alter the pathways of multipath sound scattering or give rise to microscale resonant cavities, which could contribute to improved acoustic absorption properties. The SEM images in Figure S3 show the gradual changes in the morphology and microstructure of scales on a single moth wing. Figures S3a_1_-f_1_ show the scales at low magnification, revealing a transition from wider to narrower shapes. The bottom row, Figures S3a_2_-f_2_, provides corresponding high-magnification images, detailing the surface microstructure. Through SEM observations of the moth forewings (Figure S3), we identified a naturally evolved morphological gradient: from the wing base (a) to the distal edge (f), the scales gradually become narrower and longer, while the perforation size increases and the distribution becomes denser. This gradient reflects a clear spatial functional differentiation. The broader, sparsely perforated scales at the wing base provide mechanical reinforcement, stabilizing the membrane structure and resisting fatigue during flapping, whereas the thinner, more flexible, and densely perforated scales at the edges facilitate localized air–structure interactions and energy dissipation. Such hierarchical porosity contributes to pressure equalization and viscous losses, making the distal region a naturally tuned acoustic–structural interface.


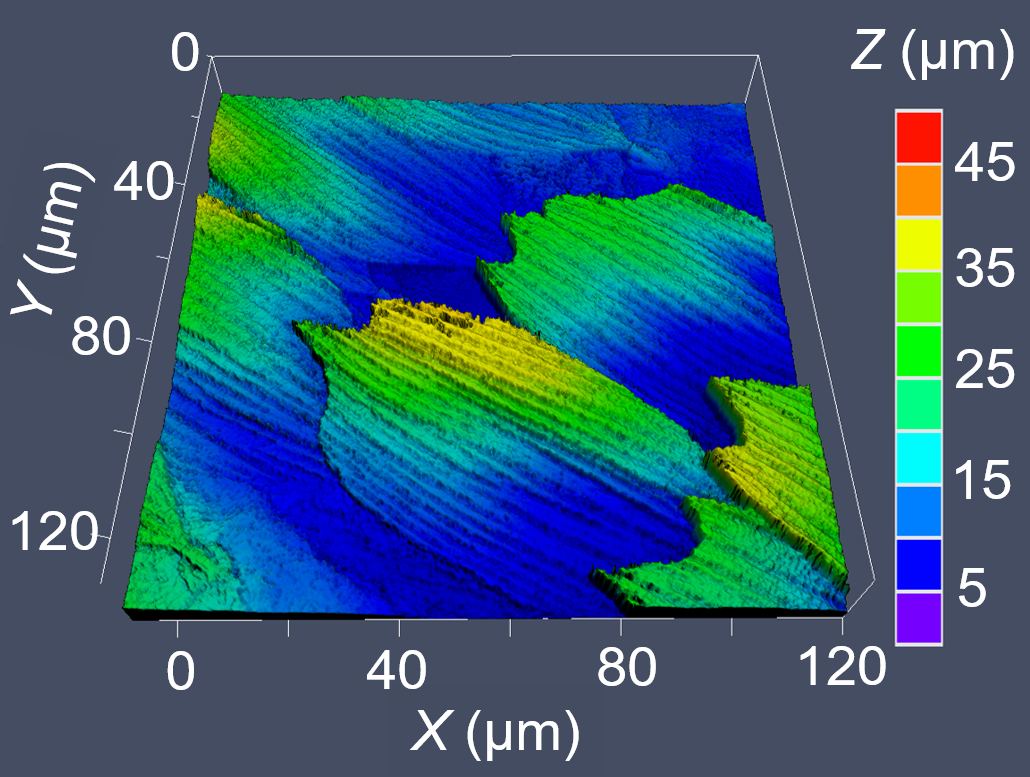


**Figure S2.** The 3D surface topography showing the scales on a moth wing.

**
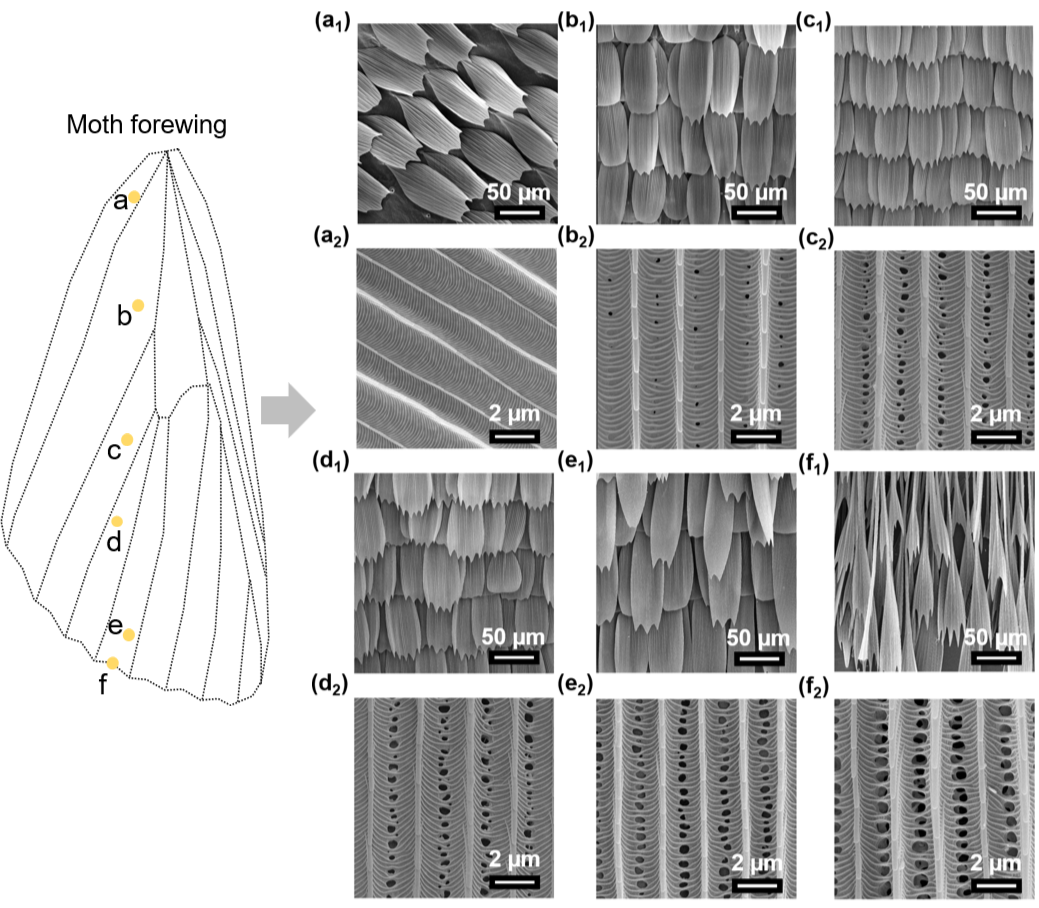
**

**Figure S3.** The SEM images showing the gradual changes in the morphology and microstructure of scales on a moth wing: (a_1_-f_1_) the SEM images of scales at low magnification and (a_2_-f_2_) the corresponding high-magnification images.

Specifically, moth wing scales have evolved hierarchical multilayered porous microstructures (Figure S4a) that generate multiple localized resonances, enabling ultrasonic absorption (typically 20–150 kHz) to evade echolocating bats.^[1]^ In contrast, butterfly scales exhibit a markedly different morphology, characterized by extremely high porosity and low material fraction (Figure S4b).^[2]^ Such structures are optimized not for acoustic damping but for structural coloration, where multi-path scattering and refractive index contrast produce vivid optical effects with minimal mass cost.^[3,4]^ This difference is evolutionarily reasonable, as butterflies are diurnal and non-phototactic species, and thus face no selective pressure for acoustic stealth.


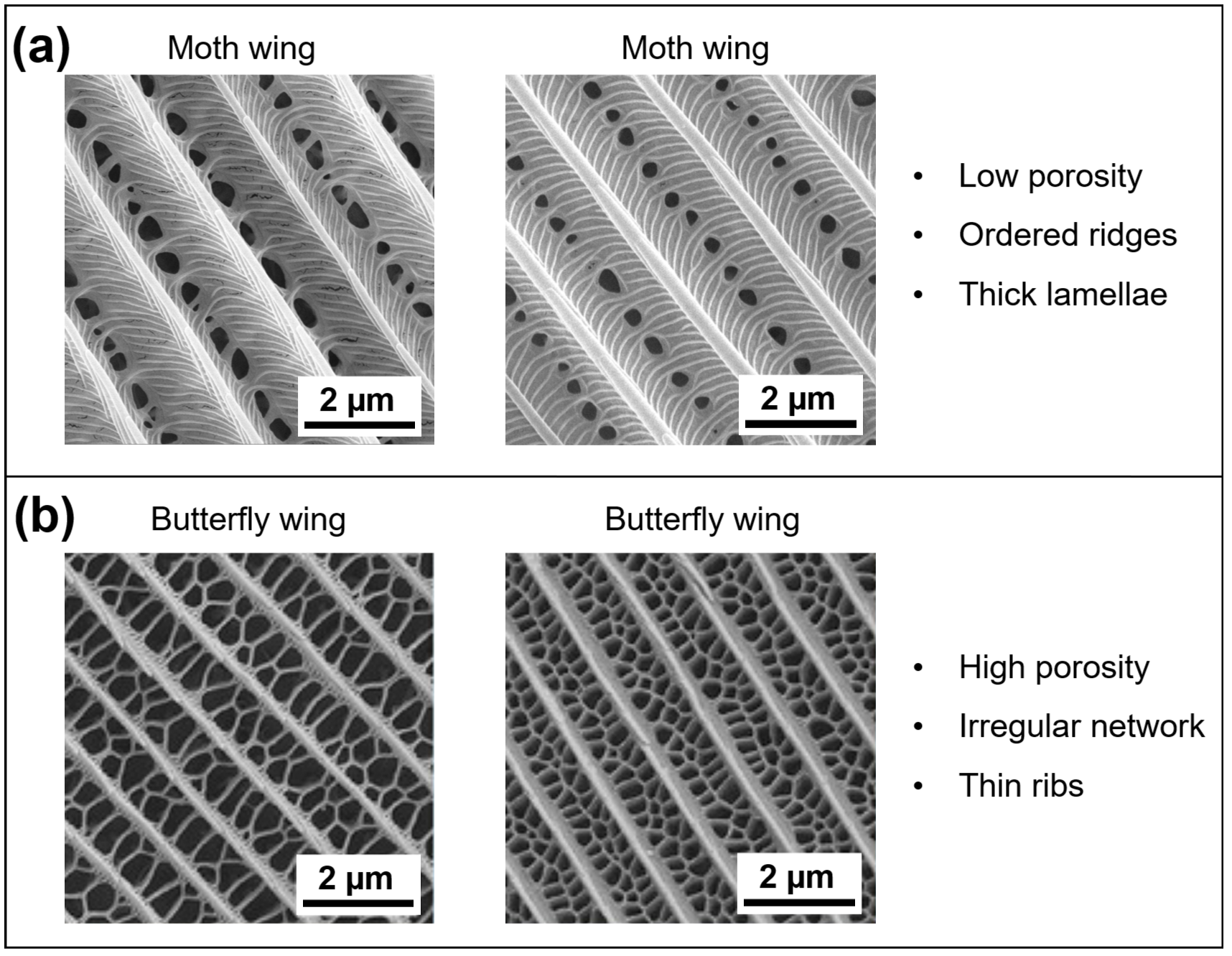


**Figure S4.** The SEM images of (a) moth wing scales and (b) butterfly wing scales.^[1]^

Beyond Lepidoptera, we also reviewed relevant literature on other insect groups such as fireflies (Coleoptera) and dragonflies (Odonata). Fireflies are nocturnal but do not rely on acoustic stealth; instead, they evolved chemical defenses and bioluminescent warning signals to deter predators.^[5,6]^ Their cuticular surfaces (elytra and hindwings) are typically covered with microscale ridges, grooves, hairs, and dome-like protrusions, which are associated with water repellence, self-cleaning ability, and enhanced mechanical strength, rather than acoustic absorption.^[7]^ By contrast, dragonfly wings display a corrugated membrane architecture supported by a dense network of longitudinal and cross veins, which provides outstanding aerodynamic stability and load-bearing capability during flight.^[8,9]^ The surface micro textures also contribute to drag reduction, anti-wetting behavior, and antibacterial properties, highlighting another pathway of natural optimization unrelated to sound absorption but valuable for bioinspired engineering.^[10]^

These comparisons reveal that the sound-absorbing microarchitecture is a unique evolutionary adaptation of moth wings, while other insect species demonstrate distinct functional optimizations in optical, mechanical, aerodynamic, or chemical aspects, driven by their ecological requirements. Such diversity enriches the field of bioinspired design by providing alternative structural motifs that can be explored for multifunctional engineering, including hydrophobicity, optical modulation, aerodynamic enhancement, and mechanical robustness.

**S3. Theoretical modeling of acoustic impedance in multilayer biomimetic sound absorber**

To quantitatively predict the acoustic performance of multilayer biomimetic sound-absorbing structures, we developed a theoretical model based on acoustic impedance theory, integrating the transfer matrix method with electroacoustic analogies. This approach enables accurate correlation between geometric pore parameters and sound absorption characteristics, providing a robust framework for the design and optimization of porous acoustic metamaterials. The model is constructed upon the classical theory of perforated plate absorbers and considers the combined effects of individual pore resistance and cavity coupling. The MLHR configuration is characterized by two primary components: the narrow perforations and the underlying cavities, represented by the transfer matrices *T_P_* and *T_C_*, respectively.^[11]^

The transfer matrix *T_P_* for the narrow pores is related to the acoustic impedance *Z_P_* and is given by:

$T_{P}=\left[ \begin{matrix} 1 & Z_{P} \\ 0 & 1 \end{matrix} \right]$ (1)

where *Z_P_* is the acoustic impedance of the perforations, derived as:^[12]^

$Z_{p}=\frac{1}{\emptyset}(i\omega\rho_{e}t+2\tau R_{s}+i\omega\rho_{0}\delta d)$ (2)

Here, *Φ* is the surface porosity, typically calculated as *Φ* = п*d*^2^/2*w*^2^, *ω* is the angular frequency, *ρ*_0_ is the density of air (1.21 kg/m^3^), *R_s_* is the surface resistance associated with the oscillatory viscous airflow along the walls and edges of a perforation. *i* is the imaginary unit, *δ* and *τ* are empirical correction factors used to match the non-standard multi-layer Helmholtz resonators. The effective air density *ρ_e_* is given by:

$\rho_{e}=\rho_{0}\left( 1+\frac{\sigma\emptyset}{i\omega\rho_{0}}G_{c} \right)$ (3)

where *σ* is the flow resistivity, which depends on the pore shape and hydraulic radius, and is given by:

$\sigma=\frac{8\eta}{\emptyset\left( \frac{d}{2} \right)^{2}}$ (4)

Here, $\eta$ is the dynamic viscosity of air. Additionally, the correction factor *G_c_* accounts for viscous losses and is expressed as:

$G_{c}=\frac{-\frac{s}{4}\sqrt{-i}\frac{J_{1}\left( s\sqrt{-i} \right)}{J_{0}\left( s\sqrt{-i} \right)}}{1-\frac{2}{s\sqrt{-i}}\frac{J_{1}\left( s\sqrt{-i} \right)}{J_{0}\left( s\sqrt{-i} \right)}}$ (5)

where *J*_0_ and *J*_1_ are the zero-order and first-order Bessel functions, and *s* is the characteristic viscous length, given by:

$s=C\sqrt{\frac{8\omega\rho_{0}}{\sigma\emptyset}}$ (6)

with *C* being a shape-dependent factor. The end correction term 2*τR_s_* in Eq. (2) is used to represent the air friction caused by the oscillatory viscous flow at the surface of the perforated plate. This term accounts for the additional resistance to airflow due to the interaction between the air and the surface of the perforation. The expression for *R_s_* is given by:

$R_{s}=\frac{\sqrt{2{\eta\rho}_{0}\omega}}{2}$ (7)

The parameter *τ* represents the resistance end correction factor, which accounts for the adjustment in airflow resistance as sound waves propagate through narrow apertures. The factor varies with the geometric parameters of the pore, such as its diameter, since the airflow characteristics are influenced by changes in pore size and other related factors. In addition to the resistance correction, the term of i*ωρ*_0_*δd* in Eq. (2) is used to account for the additional air mass (referred to as "added air mass") at both ends of the hole when the sound waves pass through it. The correction factor *δ* is involved in the reactance end correction, which compensates for the extra vibration depth that extends outward from the perforation. As sound propagates through the hole, the vibrating air column stretches beyond the inlet and outlet, modulating the effective resonance characteristics and contributing to the total acoustic impedance. With increasing pore diameter, the ratio of the viscous boundary layer to the total cross-sectional area decreases, allowing smoother mass transfer and thus enhancing the added mass effect. Consequently, the value of *δ* increases with larger pores, reflecting more pronounced reactance contributions.

The end correction terms are expected to follow the dissipative trends observed in physical experiments and are inherently non-trivial functions of geometrical parameters, including pore diameter *d*, hole depth *t*, and other microstructural features. To accurately capture these dependencies, empirical correction models were developed by fitting the theoretical absorption curves to the experimental results obtained from homogeneous absorbers. The optimal end correction expressions, expressed in millimeters, are as follows:

$\tau=5d+\frac{19}{3}t+\frac{10}{3}$ (8)

For biomimetic metamaterials featuring complex internal cavity geometries, a pore-size-dependent empirical model for the added mass correction *δ* was similarly derived:

$\delta=\frac{2}{9}d+\frac{1}{3}t+\frac{7}{10}$ (9)

The acoustic properties of the cavity depend solely on its depth, *D*. The transfer matrix for the cavity (*T_C_*) is given by: The acoustic response of the cavity is governed solely by its depth *D*, and its behavior can be described using the transfer matrix *T_C_*, given by:

$T_{C}=\left[ \begin{matrix} \cos\left( k_{0}D \right) & iZ_{0}\sin\left( k_{0}D \right) \\ \frac{i\sin\left( k_{0}D \right)}{Z_{0}} & \cos\left( k_{0}D \right) \end{matrix} \right]$ (10)

Here, *k*_0_=*ω*/*c* is the acoustic wavenumber, where *c* is the speed of sound in air. For a multilayer structure consisting of *N* layers, the overall transfer matrix *T_T_* is computed as the sequential product of the perforation and cavity matrices for each layer:

$T_{T}=\prod_{i=1}^{N} T_{i}=T_{P1}\cdot T_{C1}\cdot T_{P2}\cdot T_{C2}\cdot\cdot\cdot T_{PN}\cdot T_{CN}=\left[ \begin{matrix} T_{11} & T_{12} \\ T_{21} & T_{22} \end{matrix} \right]$ (11)

The total acoustic impedance, *Z_r_*, is then obtained from the transfer matrix components as:

$Z_{r}=\frac{T_{11}}{T_{21}Z_{0}}$ (12)

Thus, the absorption coefficient under incident sound waves, $\alpha$, is calculated as:

$\alpha=\frac{4Re\left( Z_{r} \right)}{\left[ 1+Re(Z_{r}) \right]^{2}+Im{(Z_{r})}^{2}}$ (13)

**S4. Details of the COMSOL FEM model used to analyze the thermoviscous dissipation**

The COMSOL FEM simulates the impedance tube method (ASTM E1050-19) using a geometry based on the BSWA SW477 tube with a 30 mm diameter (Figure S5). The tube dimensions include a total length of 235 mm, a distance of 190 mm from the sound source to the first microphone position *P*(*x*_1_), and a 15 mm separation between the two microphones *P*(*x*_1_) and *P*(*x*_2_).

The pressure acoustics module is applied to the air-filled tube to simulate plane wave propagation. The tube walls are defined as sound-hard boundaries (zero normal acceleration), replicating the high impedance mismatch between steel and air in the physical setup. The thermoviscous acoustics module is employed for the sample to resolve viscous and thermal boundary layer losses. This module assumes isentropic acoustics in the bulk fluid and enforces no-slip boundary conditions (zero fluid velocity at sample surfaces) to capture energy dissipation mechanisms.

The total acoustic pressure at position *x* is expressed as:

$P\left( x \right)=Ae^{-ikx}+Be^{ikx}$ (14)

where *A* and *B* represent the amplitudes of the incident and reflected waves, respectively, and *k=2π/λ* is the wave number. For microphones at positions *x*_1_ and *x*_2_*,* the pressures are:

$P\left( x_{1} \right)=Ae^{-ikx_{1}}+Be^{ikx_{1}}$ (15)

$P\left( x_{2} \right)=Ae^{-ikx_{2}}+Be^{ikx_{2}}$ (16)

The transfer function *H*=*P*(*x*_2_)*/P*(*x*_1_) is calculated to relate the pressures at the two positions:

$H=\frac{Ae^{-ikx_{2}}+Be^{ikx_{2}}}{Ae^{-ikx_{1}}+Be^{ikx_{1}}}$ (17)

From *H*, the complex reflection coefficient *R*=*B*/*A* is derived as:

$R=\frac{e^{-ikx_{2}}-He^{-ikx_{1}}}{{{He}^{ikx_{1}}-e}^{-ikx_{2}}}$ (18)

The sound absorption coefficient *α* is computed via:

$\alpha=1-\left| R \right|^{2}$ (19)

A frequency domain study is conducted with a sweep across the target range to resolve the absorption spectrum. A boundary layer mesh is applied near the sample surfaces to resolve the viscous and thermal gradients. The spatial dissipation maps are generated using COMSOL's built-in ta_diss_tot function to quantify the total thermoviscous losses. Numerical convergence is verified through mesh independence tests and validation against analytical benchmarks.


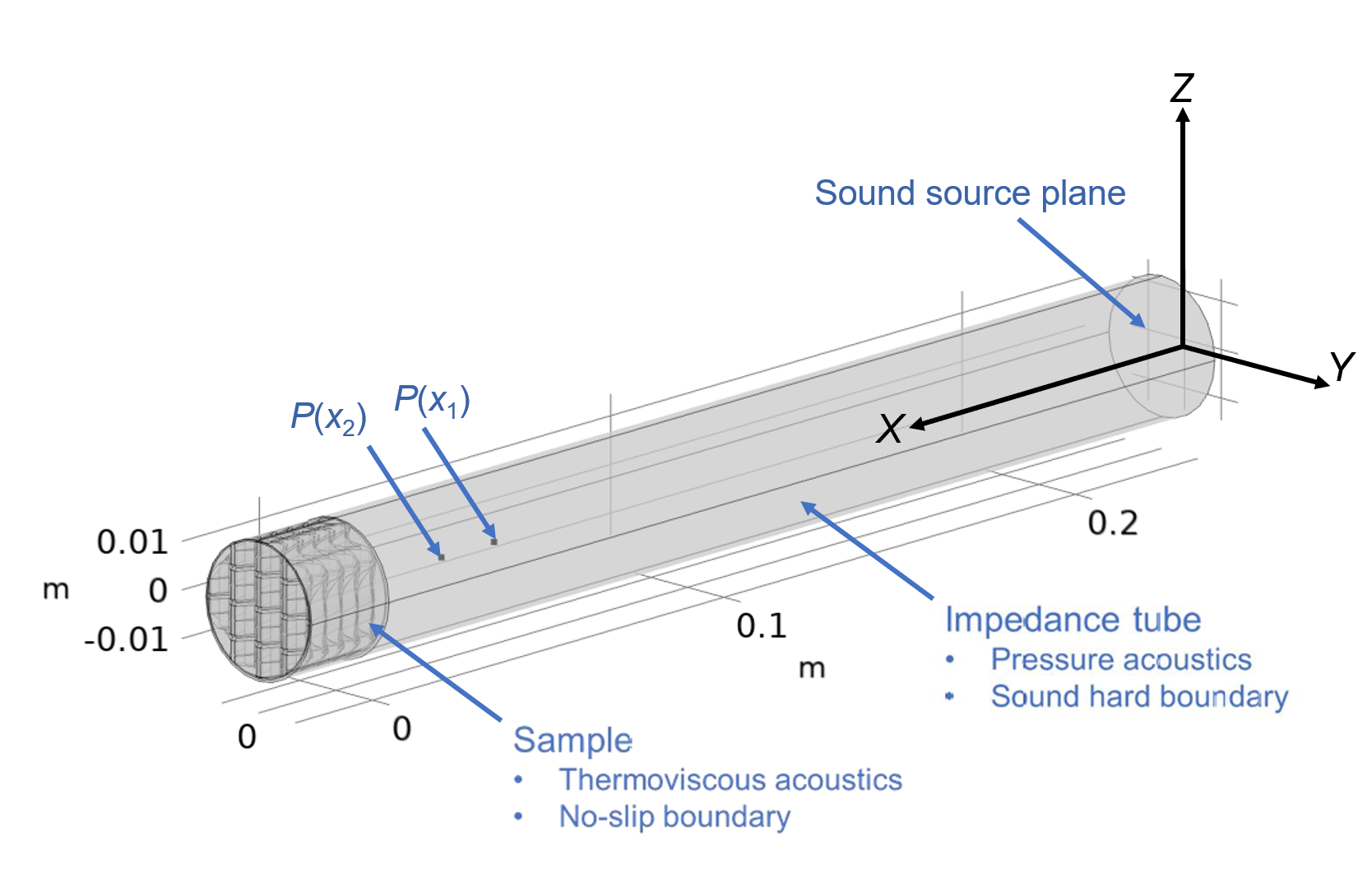


**Figure S5.** The setup and details of the COMSOL FEM model used to analyze the thermoviscous dissipation.

Figures S6 and S7 provide crucial finite element simulation insights into the micro-scale physical mechanisms underpinning the broadband sound absorption observed in the BHM. When sound waves encounter the resonant components of the BHM, the particle velocity significantly increases, indicating that the incident acoustic energy is effectively channeled into the vibrational modes. Consequently, the BHM exhibits multiple localized resonance peaks, with each peak corresponding to the specific contribution of a particular unit. Figure S6 visually corroborates this phenomenon. At various excitation frequencies, specific regions within the BHM display significantly elevated particle velocity amplitudes (yellow/orange/red regions). These high-velocity regions correspond to the particular sections of the BHM's internal structure that resonate most strongly at that specific frequency. For instance, at 5 kHz, where Figure 3d in the manuscript indicates a prominent resonance peak associated with column 4 (*d*_4_ = 1.0 mm, *t*_4_ = 1.2 mm), Figure S6 shows widespread high particle velocities within the corresponding regions, signifying efficient acoustic energy conversion into vibrational motion. This observation aligns with the principle that incident acoustic waves induce intense molecular oscillations in the confined geometries of the metamaterial.

**
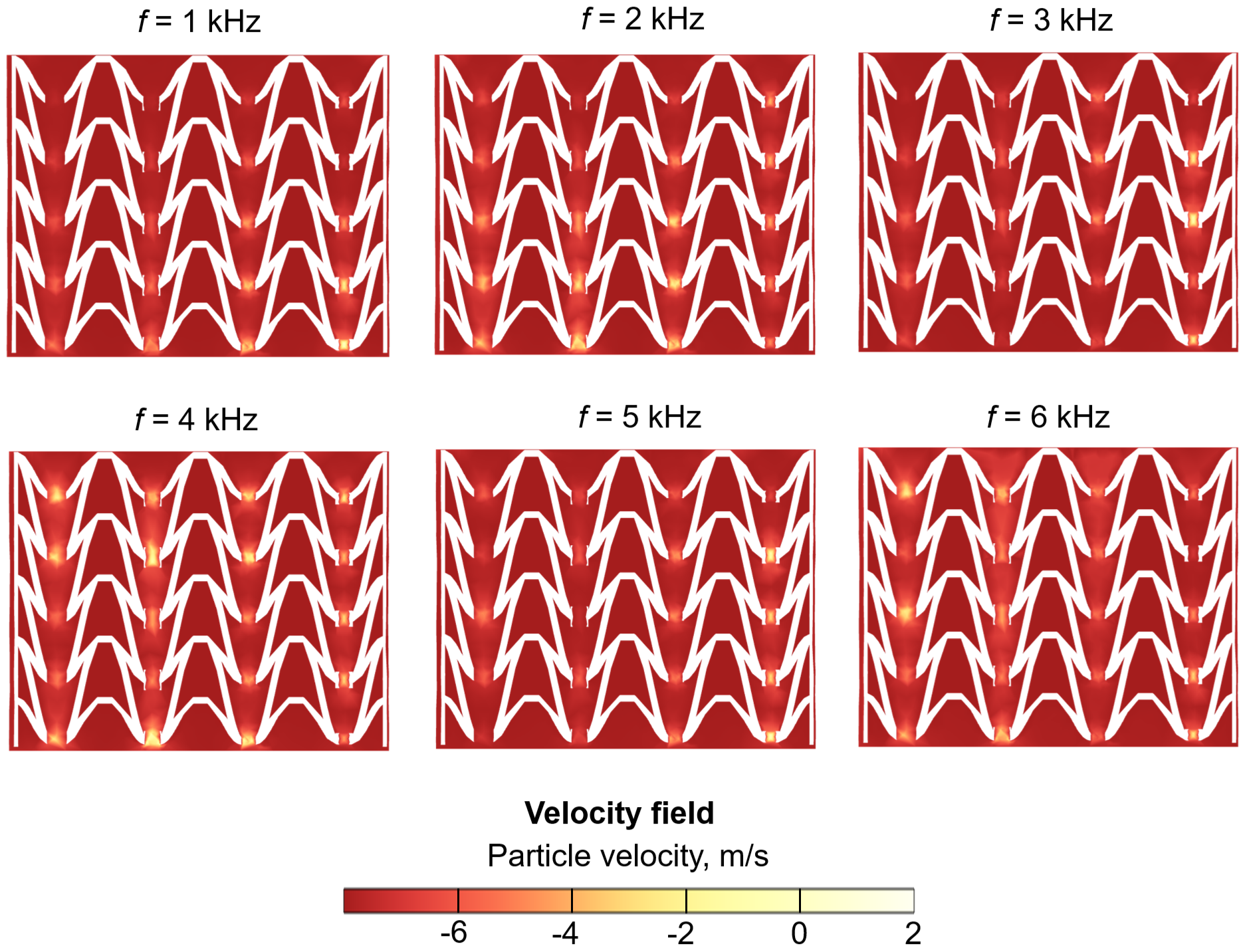
**

**Figure S6.** Finite element simulation of particle velocity field in the BHM at different frequencies.

Figure S7 illustrates the viscous energy dissipation mechanisms within the BHM through finite element simulations. The orange/red regions correspond to locations where the majority of acoustic energy is converted into heat due to thermo-viscous losses. This directly correlates with the absorption peaks observed in Figure 3f and the positions of conjugate zeros in Figure 3d. Maximum sound absorption precisely occurs at locations of highest energy dissipation, thereby confirming the dominant role of pore-resolved mechanisms in acoustic energy attenuation. Figure S7 demonstrates that significant energy dissipation occurs across a broad range of frequencies (1 kHz to 6 kHz). Rather than dissipation being confined to narrow frequency bands, Figure S7 reveals distributed areas of high dissipation across different frequencies, collectively contributing to the broadband absorption. This reinforces the concept that the heterogeneous design facilitates multiple dissipation pathways, which cumulatively result in a wide absorption spectrum.

**
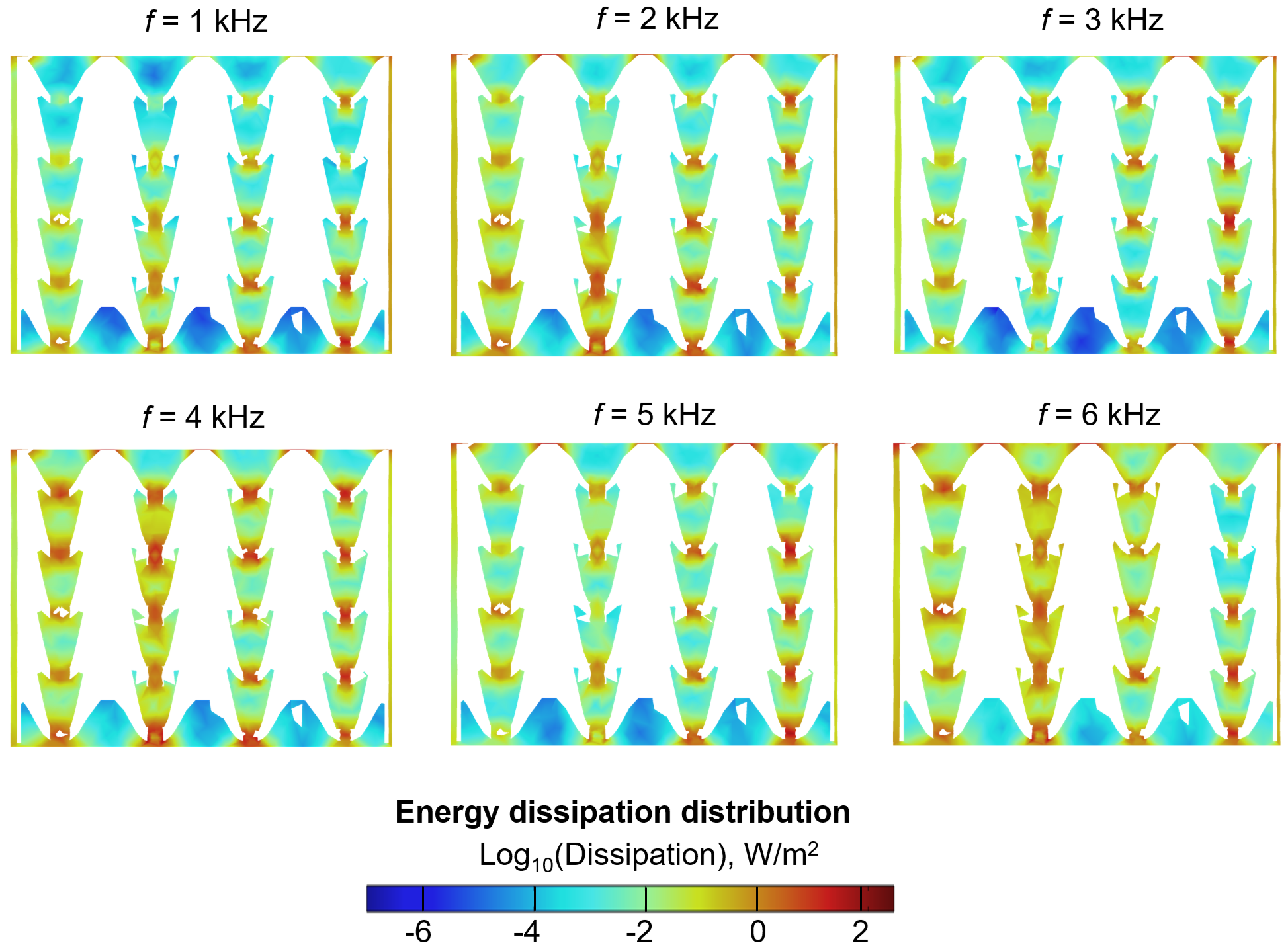
**

**Figure S7.** Finite element simulation of viscous energy dissipation distribution in the BHM at different frequencies.

Figure S8 presents the simulated distribution of thermal energy dissipation, expressed as log_10_(ta. diss_therm), to highlight the differences in dissipation mechanisms between standard micro-perforated panels and the BHM. In these simulations, viscous dissipation was not considered; the color scale exclusively reflects thermal boundary layer losses within the system.

In the standard multi-layer panel (upper panel), thermal dissipation is relatively weak and primarily confined to narrow apertures and cavity walls, with minimal enhancement from changes in sound incidence direction. In contrast, the BHM exhibits a marked increase in thermal dissipation when the direction of sound incidence is reversed. Notably, high dissipation zones concentrate at geometrically confined corners and interfaces between asymmetrically distributed cavities. These regions exhibit thermal loss intensities that are enhanced by up to two orders of magnitude, consistent with localized energy confinement and multiple internal reflections.

This simulation result reinforces that the observed enhancement in acoustic absorption under reverse excitation in the BHM does not stem from viscous mechanisms but rather from intensified thermal dissipation.

**
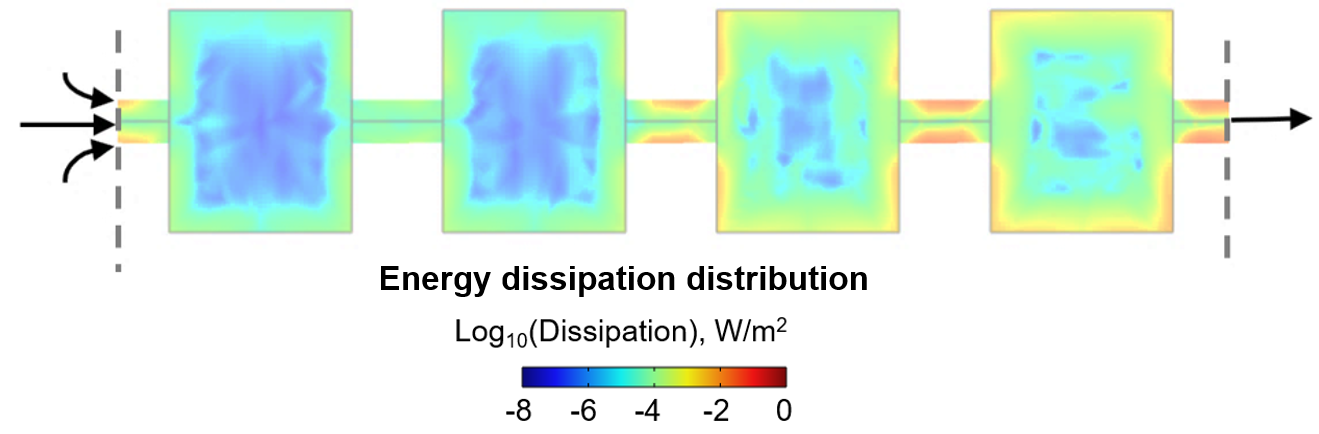
**

**Figure S8.** Finite element simulation of the thermal energy dissipation in standard micro-perforated panels.

**S5. Potential application scenarios of the developed BHM**

Potential solutions are proposed to integrate the BHM into typical noise-critical engineering scenarios, as illustrated in Figure S9. In the context of unmanned aerial vehicles (UAVs), noise generated by the rapid rotation of propellers and the operation of compact motors poses a significant challenge for both urban acoustic environments and military stealth performance. To address this, BHM structures can be wrapped around the motor housing to attenuate broadband noise while simultaneously facilitating thermal dissipation through their porous architecture. In high-speed rail systems, aerodynamic turbulence and wheel–rail interactions are major sources of low- to mid-frequency noise that degrade passenger comfort and increase environmental noise pollution. BHM can be embedded into underbody soundproof panels or interior linings to absorb and dissipate such noise efficiently.

**
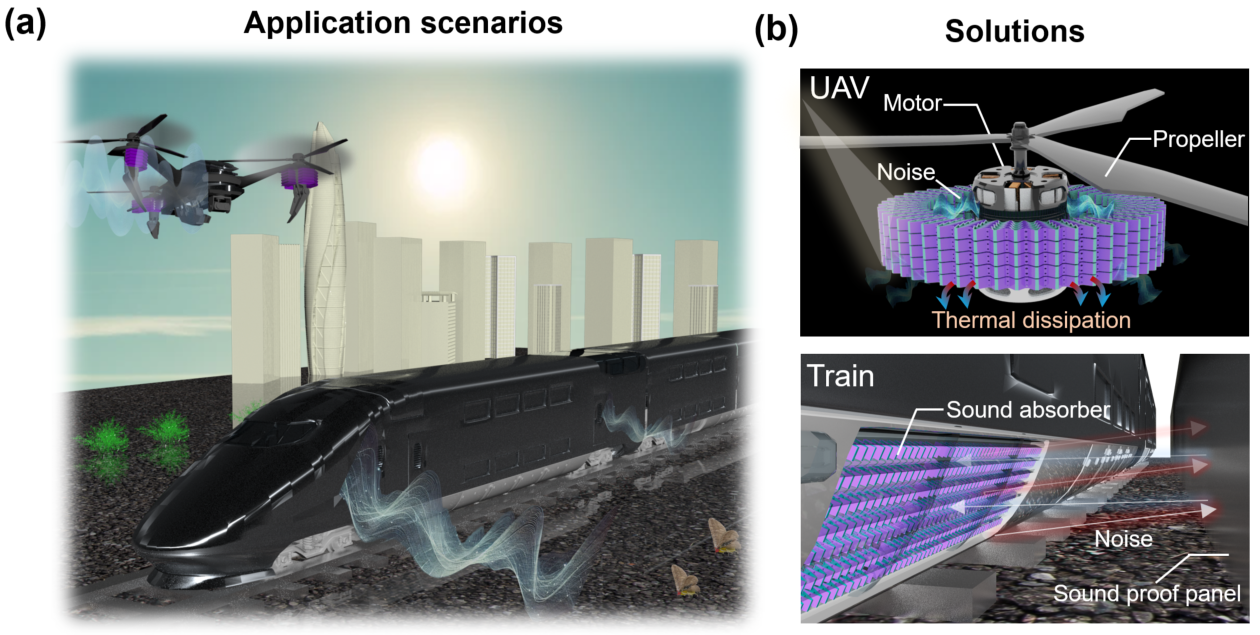
**

**Figure S9.** (a) Representative engineering scenarios and (b) BHM‑based noise‑reduction strategies.

**S6. Details of the COMSOL FEM model used to analyze the piezoelectric response of the sensor**

First, we create the geometric model (Figure S10) by simplifying the interior of the helmet. The air domain is defined by performing a Boolean difference operation between two approximately hemispherical volumes. At the center of the bottom surface of the air domain, a piezoelectric layer (0.2 mm thick) is embedded, and the top boundary of the air domain is set as an impedance boundary. The material properties are assigned. The air domain uses default acoustic parameters (density *ρ* = 1.21 kg/m³, speed of sound *c* = 343 m/s).

For the piezoelectric material (PZT-5H), the piezoelectric coupling matrix is defined as:

{0[C/m²], 0[C/m²], 0[C/m²], 0[C/m²], 17.0345[C/m²], 0[C/m²], 0[C/m²], 0[C/m²], 0[C/m²], 17.0345[C/m²], 0[C/m²], 0[C/m²], -6.62281[C/m²], -6.62281[C/m²], 23.2403[C/m²], 0[C/m²], 0[C/m²], 0[C/m²]},

and the elastic stiffness matrix is set as:

{1.27205e+11[Pa], 8.02122e+10[Pa], 8.46702e+10[Pa], 0[Pa], 0[Pa], 0[Pa], 8.02122e+10[Pa], 1.27205e+11[Pa], 8.46702e+10[Pa], 0[Pa], 0[Pa], 0[Pa], 8.46702e+10[Pa], 8.46702e+10[Pa], 1.17436e+11[Pa], 0[Pa], 0[Pa], 0[Pa], 0[Pa], 0[Pa], 0[Pa], 2.29885e+10[Pa], 0[Pa], 0[Pa], 0[Pa], 0[Pa], 0[Pa], 0[Pa], 2.29885e+10[Pa], 0[Pa], 0[Pa], 0[Pa], 0[Pa], 0[Pa], 0[Pa], 2.34742e+10[Pa]}.

The density *ρ* is set to 7500 kg/m³, and the polarization direction is confirmed to be along the thickness direction (Z-axis).

In the physics setup, the air domain is assigned the pressure acoustics module with an incident plane wave as the acoustic source. For the piezoelectric domain, solid mechanics and electrostatics interfaces are assigned, and the piezoelectric effect is enabled via a multiphysics coupling node. On the piezoelectric layer, the lower surface is set to ground (0 V) and the upper surface is defined as a terminal boundary. Finally, an acoustic-structureboundary condition is applied at the interface between the air and the piezoelectric material to realize the coupling between sound pressure and mechanical displacement.

**
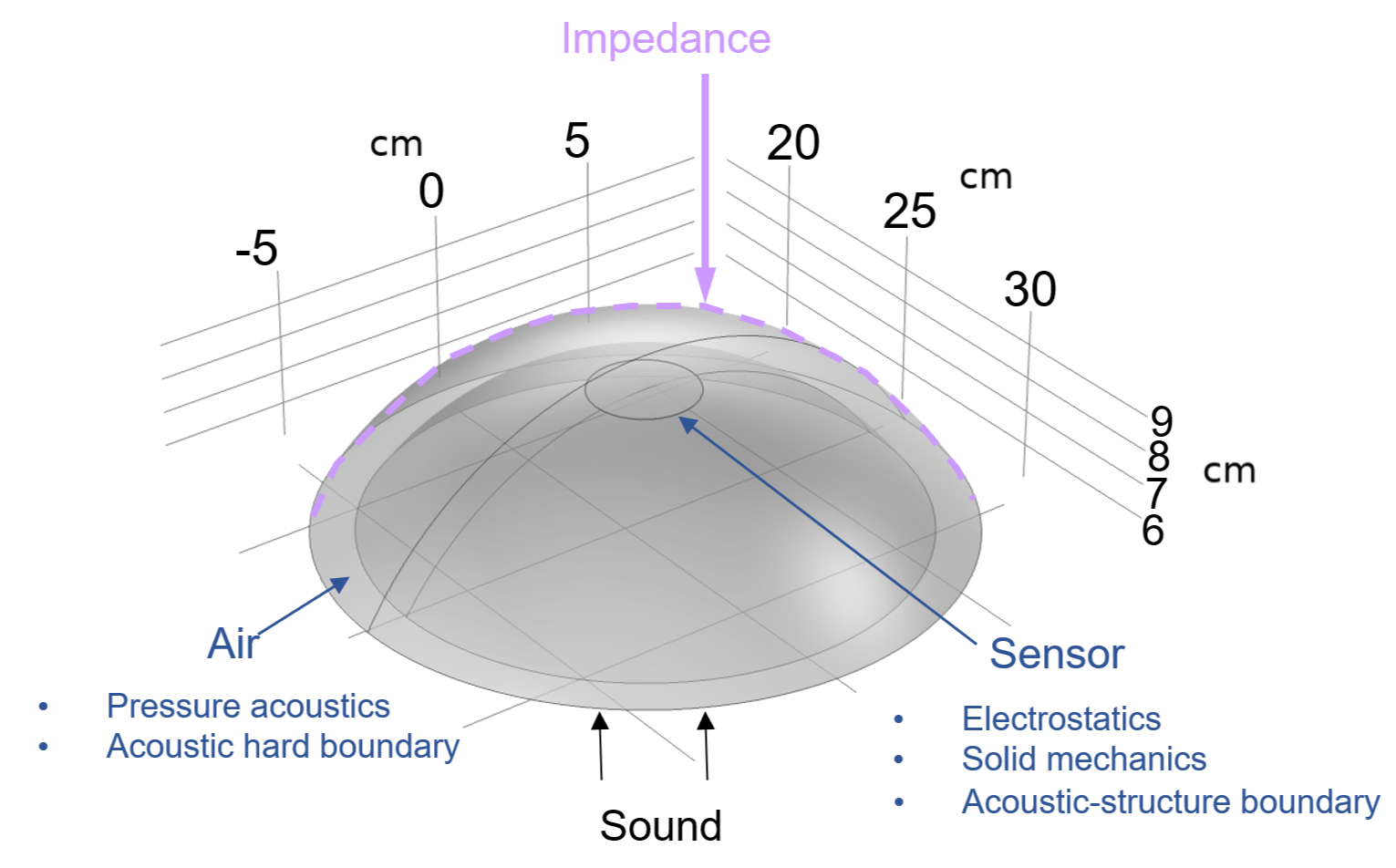
**

**Figure S10.** The details of the COMSOL FEM model used to analyze the piezoelectric response of the sensor.

**
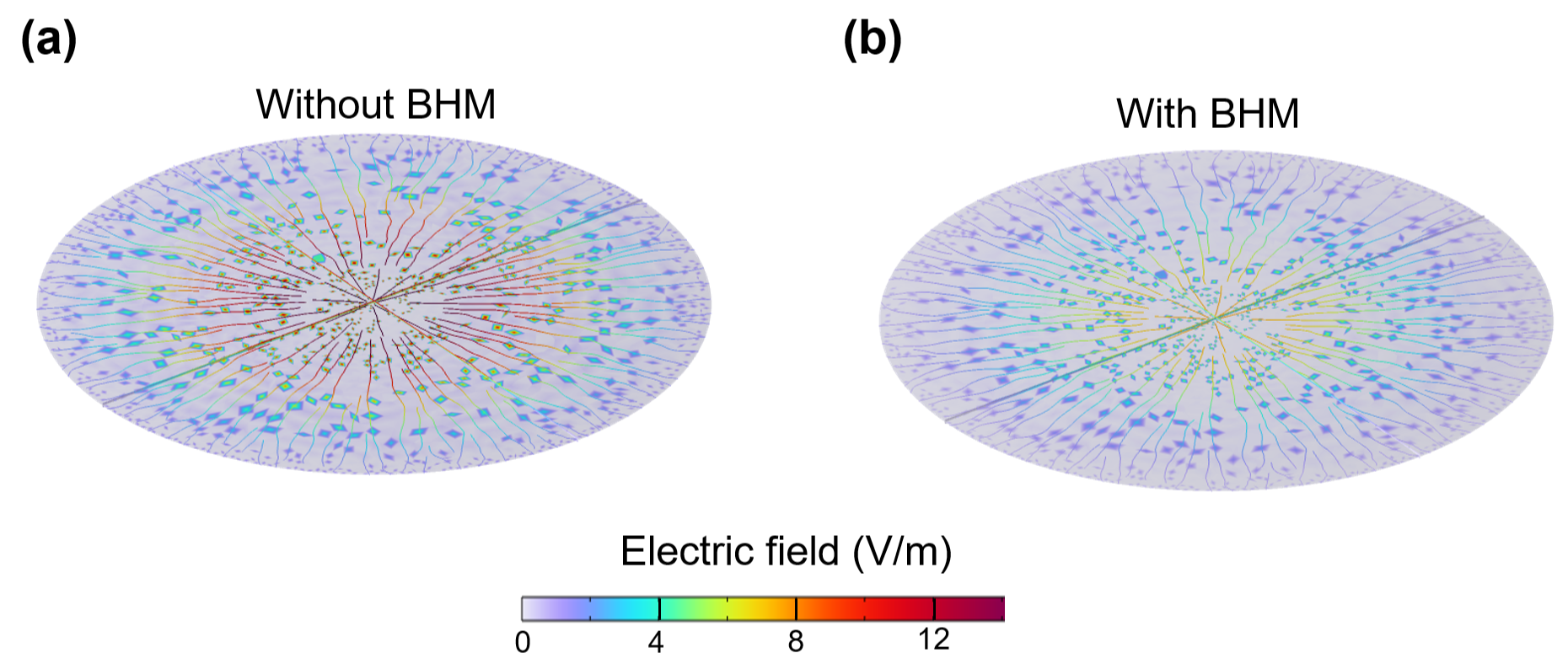
**

**Figure S11.** COMSOL simulations of the electric‑field distribution in the helmet‑mounted piezoelectric sensor: (a) sensor without a backing layer and (b) sensor backed by the BHM liner.

**
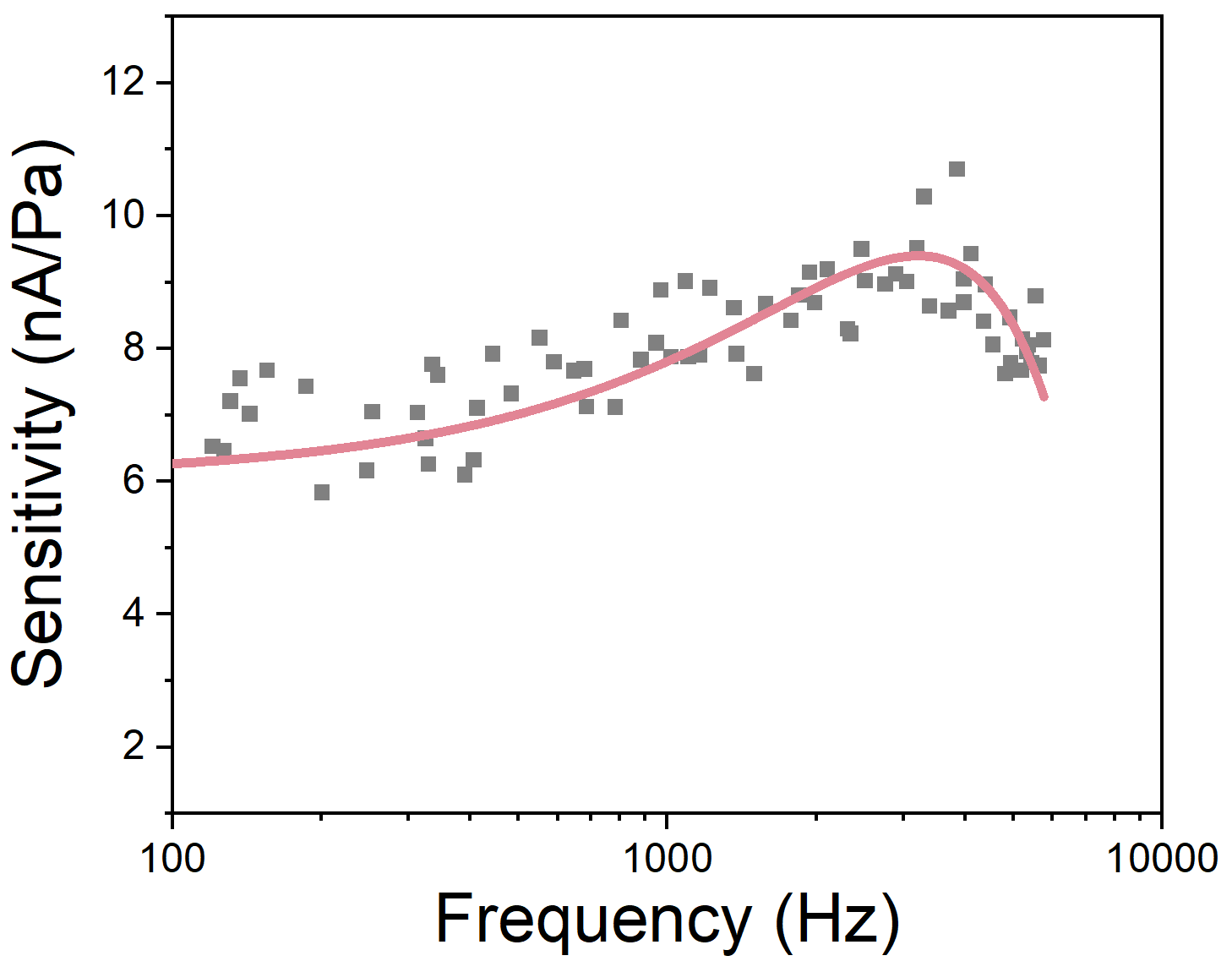
**

**Figure S12.** The acoustic frequency response of the PZT sensor measured under the blank helmet configuration.

**
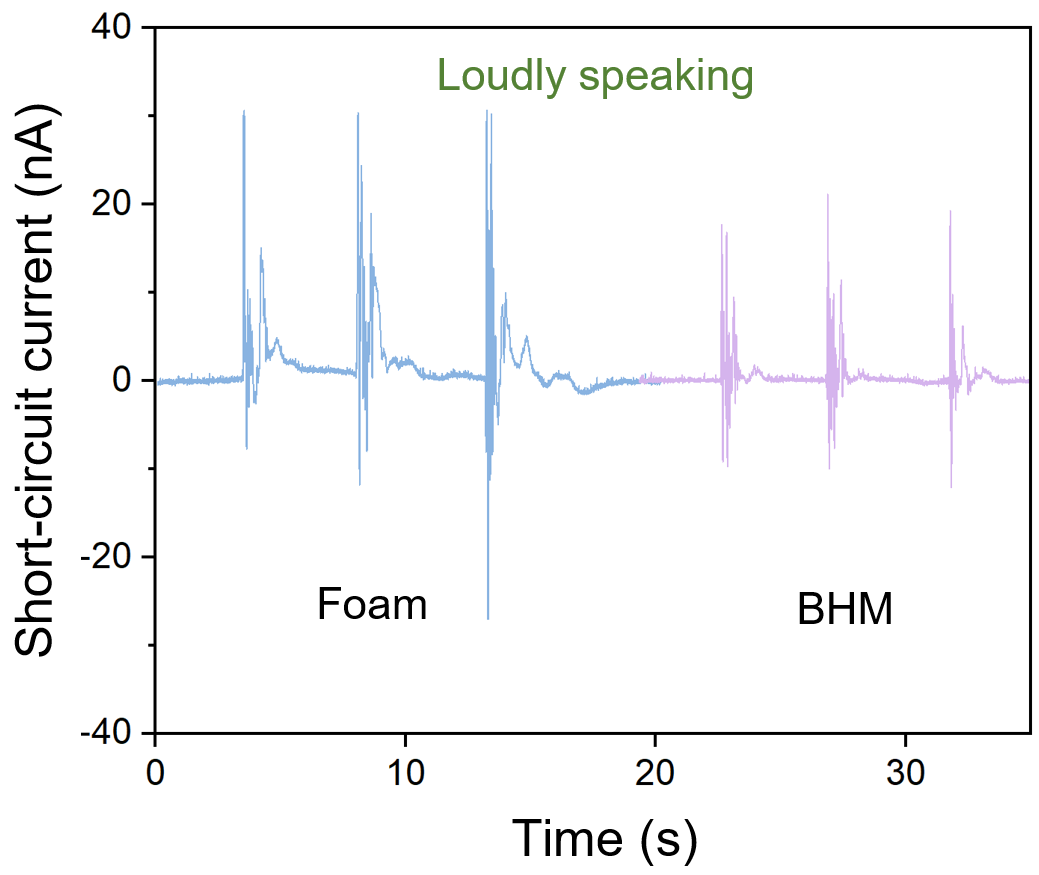
**

**Figure S13.** Short-circuit current responses of the PZT sensor under acoustic excitation from loud human speech.

**S7. Details of the Abaqus model used to analyze the** **mechanical energy dissipation**

Abaqus was utilized to investigate the mechanical response of the BHM under compressive loading. The structure was discretized using 10-node modified quadratic tetrahedral elements (C3D10M), and after mesh convergence the model comprised 14,092,683 elements and 20,054,890 nodes, which were directly imported from the CAD model created in SOLIDWORKS. The elastic–plastic constitutive model with isotropic hardening was employed for both the elastic and plastic regions, using the von Mises yield criterion to capture the post-yield behavior. General contact (Explicit) was activated for all exterior faces with self-contact enabled. The global interaction property IntProp-1 employed a penalty-based tangential formulation with an isotropic friction coefficient μ = 0.25 (no slip-rate, pressure-, or temperature-dependent terms), while the normal behavior was set to “hard” pressure-overclosure with separation allowed, using the default constraint-enforcement method. Then a mesh convergence study was conducted to ensure that further refinement had minimal impact on the numerical results. The top plate was modeled as a discrete rigid body associated with a reference point (F_top) to track the time history of the reaction forces. A displacement load was applied in the negative z-direction at this reference point while all other degrees of freedom were fixed; meanwhile, the bottom plate was completely constrained. A single Dynamic, Explicit step with large-deformation kinematics drove the analysis. No mass scaling was introduced, and the kinetic-to-internal energy ratio stayed below 3 %, validating quasi-static conditions in the explicit framework.

Figure S14 illustrates the progressive crushing of the BHM lattice at strains exceeding 30 %. Finite-element von Mises stress contours (upper sub-rows; color scale 0–105 MPa at right) are juxtaposed with synchronized optical images from the compression test (lower sub-rows). The first triplet of columns documents the transition from uniform elastic bending to the onset of layer-wise buckling and lateral re-entrance and finally to pronounced local collapse. The second triplet extends the sequence into the densification regime: by *ε* ≈ 45 % most concave struts have folded, by ε ≈ 55 % inter-strut contact dominates, and by *ε* ≈ 65 % the lattice has compacted into a stress-bearing block with peak stresses exceeding 100 MPa. The close agreement in deformation mode and stress localization across all stages confirms that the explicit elastoplastic model faithfully reproduces the full multistep compression mechanism observed experimentally.

**
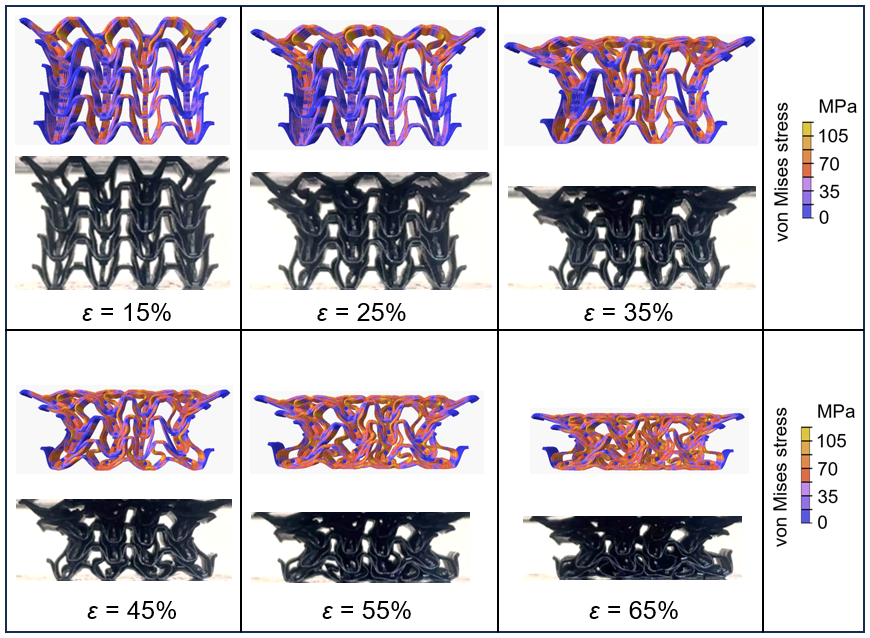
**

**Figure S14.** Comparison between the Abaqus simulation and digital images of the BHM during compression, which accurately reproduces initial elastic bending of ligaments, layer-wise buckling and stress redistribution, and final densification with contact-controlled hardening.

**S8. Digital Image Correlation (DIC) Methodology for Displacement Analysis of BHM**

DIC was employed to quantify the full-field displacement of the samples during mechanical testing. This non-contact optical technique involves applying a high-contrast random speckle pattern, with an average diameter of 0.2 mm, to the surface of each sample (Figure S15). During the experimental procedures, high-definition cameras continuously captured images of the deforming speckle pattern in real-time. Subsequently, these sequential images were analyzed using commercial Digital Image Correlation software (MATLAB, MathWorks Inc., USA). The software cross-correlated subsets of pixels within the images to accurately track the displacement of the speckles, thereby generating high-resolution displacement contour maps. These maps provided detailed quantitative data for further computational analysis of the sample's deformation behavior.

**
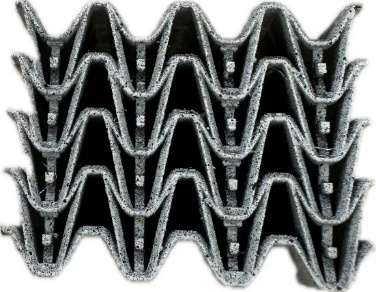
**

**Figure S15.** The digital image of the sample prepared for digital image correlation (DIC) experiments.

**
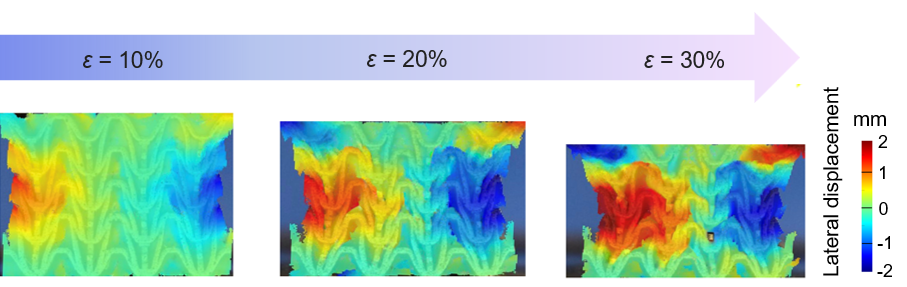
**

**Figure S16.** Lateral displacement results of the BHM obtained via DIC.

**S9. The theoretical method for calculating the effective thermal conductivity**

For low-temperature thermal insulation applications, radiation heat transfer is neglected because the Stefan–Boltzmann contribution is several orders of magnitude smaller than the conductive term for the present *ΔT* ≤ 50 K.^[13,14]^ The total heat flux due to conduction is divided into two components, contributed by the plates and rods, respectively. Invoking Fourier's first law for a homogeneous element of thickness *S* and cross-sectional area *A*, the heat flux *Φ*_1_ (in watts) generated by the two-dimensional plates can be calculated using Fourier's law, assuming the heat transfer process reaches a steady state after thermal equilibrium is achieved:^[15]^

$\Phi_{1}=A_{1}\lambda_{0}\frac{T_{2}-T_{1}}{S_{1}}$ (20)

where *λ*₀ (mW·m⁻¹·K⁻¹) denotes the intrinsic thermal conductivity of the base polymer. Based on the geometric characteristics of the lattice structure, the conductive area *A_1_* and the thermal conduction path *S*_1_ of the two-dimensional plates can be expressed as follows:

$S_{1}=m(L_{1}+L_{2})$ (21)

Here, the equations for *L*₁ and *L*₂ are derived using the complete elliptic integral of the second kind, *E*(*k*), which accounts for the curved heat-flow trajectory within the re-entrant cell walls, to determine the thermal conduction path length of the two-dimensional plates:

$L_{1}=\frac{2l}{\pi}\sqrt{1+\left( \frac{h_{1}\pi}{2l} \right)^{2}}\cdot E\left( \frac{\frac{h_{1}\pi}{2l}}{\sqrt{1+\left( \frac{h_{1}\pi}{2l} \right)^{2}}} \right)$ (22)

$L_{2}=\frac{2l}{\pi}\sqrt{1+\left( \frac{h_{2}\pi}{2l} \right)^{2}}\cdot E\left( \frac{\frac{h_{2}\pi}{2l}}{\sqrt{1+\left( \frac{h_{2}\pi}{2l} \right)^{2}}} \right)$ (23)

$A_{1}=ntw$ (24)

where *n* is the total number of repeating units in both the width and length directions, *m* is the number of repeating units in the thickness direction, and *t* and *w* represent the wall thickness and cell width, respectively.

The heat flux *Φ*_2_ contributed by the rods, as well as their conductive area *A*_2_ and thermal conduction path *S*_2_, can be expressed as follows:

$S_{2}=m\frac{(h_{1}-h_{2})}{sin\alpha}$ (25)

$A_{2}=2n{t_{1}}^{2}$ (26)

$\Phi_{2}=A_{2}\lambda_{0}\frac{T_{2}-T_{1}}{S2}$ (27)

In Eqs. (25)-(27), the slender diagonal rods are treated as prismatic bars inclined at an angle α to the heat-flow direction, so their effective conductive length along the temperature gradient is (*h*₁ − *h*₂)/sin*α*, while their cross-sectional area is approximated by twice the square of the rod side length *t*₁. Furthermore, the lattice structure can be approximated as an equivalent solid plate, with an effective heat flux *Φ_r_* given by:

$\Phi_{r}=A_{r}\lambda_{r}\frac{T_{2}-T_{1}}{mh_{1}}$ (28)

where *A_r_* (mm²) is the cross-sectional area of the equivalent plate, *λ_r_* (mW·m^−1^·K^−1^) is the effective thermal conductivity of the lattice.

$\lambda_{r}=\lambda_{0}\cdot\frac{h_{1}}{w^{2}}\left[ \frac{t}{\frac{2l}{\pi}\sqrt{1+\left( \frac{h_{1}\pi}{2l} \right)^{2}}\cdot E\left( \frac{\frac{h_{1}\pi}{2l}}{\sqrt{1+\left( \frac{h_{1}\pi}{2l} \right)^{2}}} \right)+\frac{2l}{\pi}\sqrt{1+\left( \frac{h_{2}\pi}{2l} \right)^{2}}\cdot E\left( \frac{\frac{h_{2}\pi}{2l}}{\sqrt{1+\left( \frac{h_{2}\pi}{2l} \right)^{2}}} \right)}+\frac{2{t_{1}}^{2}sin\alpha}{h_{1}-h_{2}} \right]$ (29)

**S10. Strain-dependent acoustic performance of the BHM**

For our negative Poisson's ratio structure, deformation is confined within reduction of the layer height, while the horizontal feature does not generally deform. Thus, with increasing mechanical deformation, for the acoustic properties, cavity height (*D*) reduces proportionally to strain while pore sizes (*d*) and thickness (*t*) remain generally the same. Thus, changes to the sound absorption behavior would be primarily related to reduction in *D*, with small variations to *d* and *t*. The expected sound absorption behavior could be predicted using Equations (1) – (13). Generally, for full-strain recovery in a pristine structure, no changes to sound absorption curves would be expected since there is no change to the structure. However, after multiple loading cycles, due to accumulated damage and hysteresis, the structure can only partially recover. Thus, herein, we also perform a detailed experimental investigation to show the influence of different strain-recovery level on the sound absorption. Figure S17 shows the super-depth microscope images of both central and edge pores under 0% and 8% plastic strain, revealing markedly different deformation behaviors. To provide clarity for reproducibility, the typical manufacturing tolerances of our DLP-printed samples were verified: the dimensional deviation of the pore diameter *d* was within ±0.02 mm, and the overall variation in porosity remained within ±0.1%, ensuring that the observed strain-dependent effects originate from mechanical deformation rather than fabrication variability. Due to the negative Poisson's ratio effect, the pores near the central region underwent noticeable contraction accompanied by an increase in depth, whereas the edge pores experienced lateral stretching, transforming their circular openings into elliptical shapes. This spatially nonuniform deformation redistributes the cavity volume and alters the local resonance conditions.


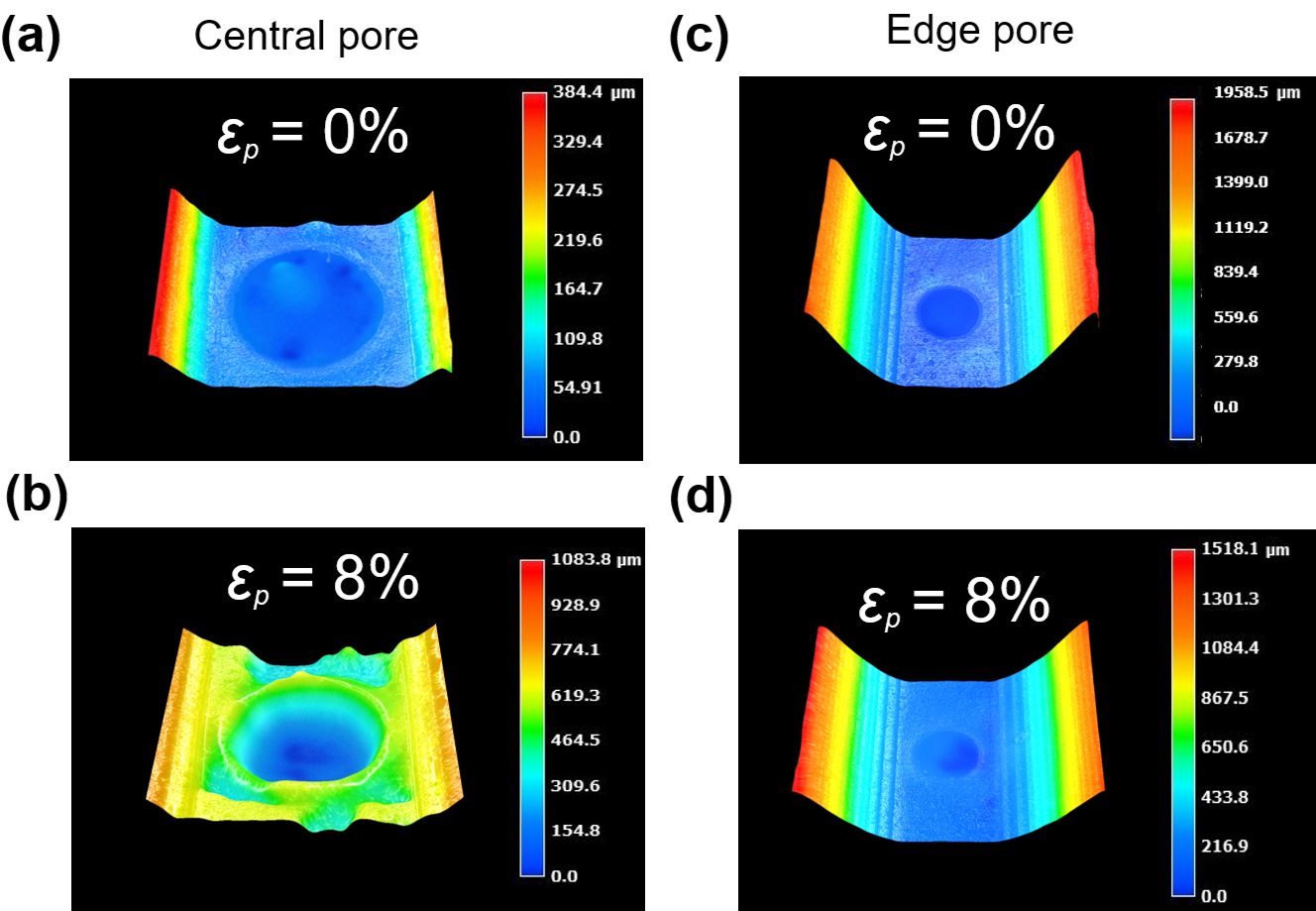


**Figure S17.** The super-depth microscope images showing the morphological evolution of central (a, b) and edge (c, d) pores at different plastic strains (*ε_p_*= 0% and 8%).

The corresponding absorption spectra for the deformed samples are shown in Figure S18 (the black curves indicate the undeformed state). At small plastic strains (*ε_p_* ≈ 3%), the applied load was primarily transferred through the concave hexagonal interlayer struts, which distributed stress uniformly. The negative Poisson's ratio led to lateral expansion and slight upward bending at the top edges, thereby inducing a modest redistribution of the adjacent back-cavity volume and slightly increasing near the outer region while the lower cavities remained largely unchanged. The pore size remained almost unchanged. As a result, the main resonance frequency exhibited almost no shift.

When the strain increased to *ε_p_* ≈ 5%, the overall structural thickness decreased, and convergence of the mid-lower cells reduced the effective cavity volume. Although the top pores continued to stretch, increasing flow resistance and restricting high-frequency mode transmission. Consequently, the absorption spectrum showed attenuation in the high-frequency range (4500–6000 Hz) and a slight rightward shift of the primary resonance peak, indicating the onset of geometric densification.

At a higher strain of *ε_p_* ≈ 8%, the interlayer struts became unstable and partially collapsed, entering a stage of geometric compaction. The cavity depth and effective compliance were significantly reduced, and the overall stiffness increased, causing the main resonance frequency to shift toward higher frequencies. Simultaneously, pore-neck narrowing and minor edge cracking further increased the end correction and flow resistance, degrading impedance matching and reducing the absorption peak. In summary, the BHM exhibits a strain-dependent acoustic response governed by the interplay between pore deformation, cavity volume evolution, and viscous loss.


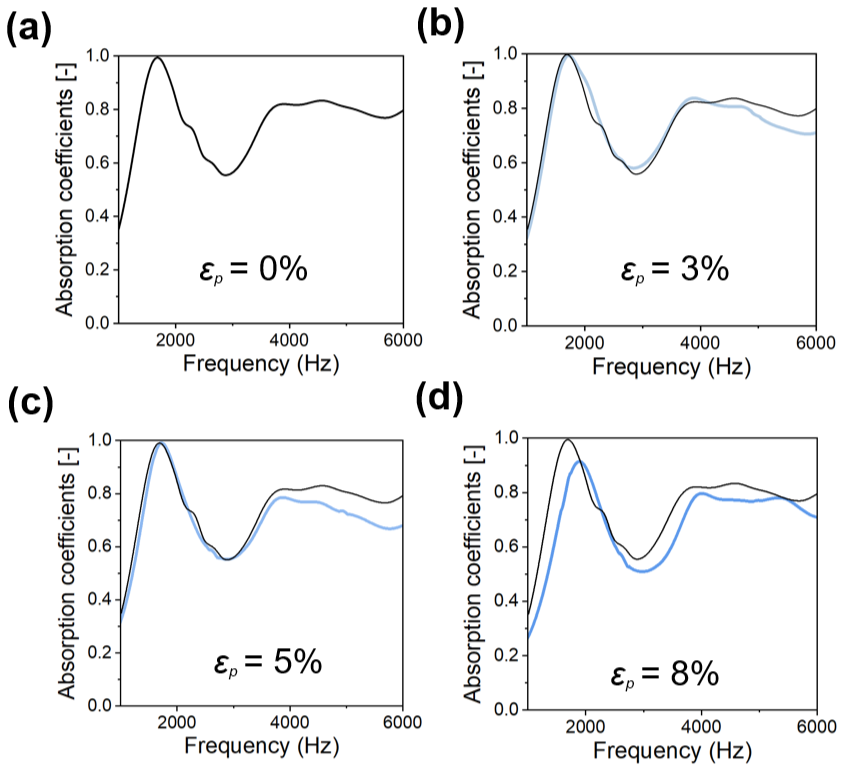


**Figure S18.** The absorption coefficient curves of BHM samples under plastic strains of (a) 0%, (b) 3%, (c) 5%, and (d) 8%.

**S11. Long-term stability of the BHM under cyclic and hygrothermal conditions**

To evaluate the long-term durability of the BHM, additional experiments were conducted focusing on cyclic fatigue and hygrothermal aging.

Under a strain rate of 0.01 s⁻¹ and a strain amplitude of 10%, the engineering stress gradually decreased over 2000 compression cycles due to slow recovery and irreversible structural damage (Figure S19a). After allowing the sample to recover naturally for 12 h, acoustic measurements revealed a reduced absorption peak and a rightward shift of the main resonance frequency (Figure S19b). This phenomenon can be attributed to stress concentration–induced microcrack formation along the pore edges, which increased the effective porosity and leakage paths, thereby disturbing the original impedance matching and leading to attenuation and frequency shift of the main absorption peak. In principle, the proposed metamaterial can be made flexible by modifying the photopolymer resin composition, such as introducing soft or elastomeric monomers (e.g., polyurethane acrylate, butyl acrylate). This can significantly enhance mechanical compliance and adaptability while maintaining the designed geometry, improve formability on complex curved substrates, and mitigate damage accumulation (e.g., brittleness and stress-concentration–induced microcracks at pore edges), thereby enhancing crack tolerance and cyclic durability.

To assess environmental stability, alternating hygrothermal aging tests (30–60 °C, 85–95% relative humidity, 24 h per cycle for 10 cycles) were performed to evaluate the effects of moisture and temperature. As shown in Figure S19c, the mechanical modulus decreased, indicating softening after moisture uptake. The yield point shifted toward lower strain, and the overall strength declined. For the acoustic response, moisture absorption softened the resin matrix and slightly reduced structural stiffness, leading to a lower absorption peak at low frequencies, while the resonance frequency remained nearly unchanged (Figure S19d). The acrylate-based resin used in this study contains polar functional groups (C=O, C–O–C, and –OH), which exhibit moderate hygroscopicity. To mitigate moisture sensitivity, several material-level strategies can be adopted, such as introducing hydrophobic monomers (e.g., fluorinated acrylates), applying fluorosilicone surface coatings,^[16]^ or employing plasma-induced hydrophobic surface treatments. ^[17]^

**
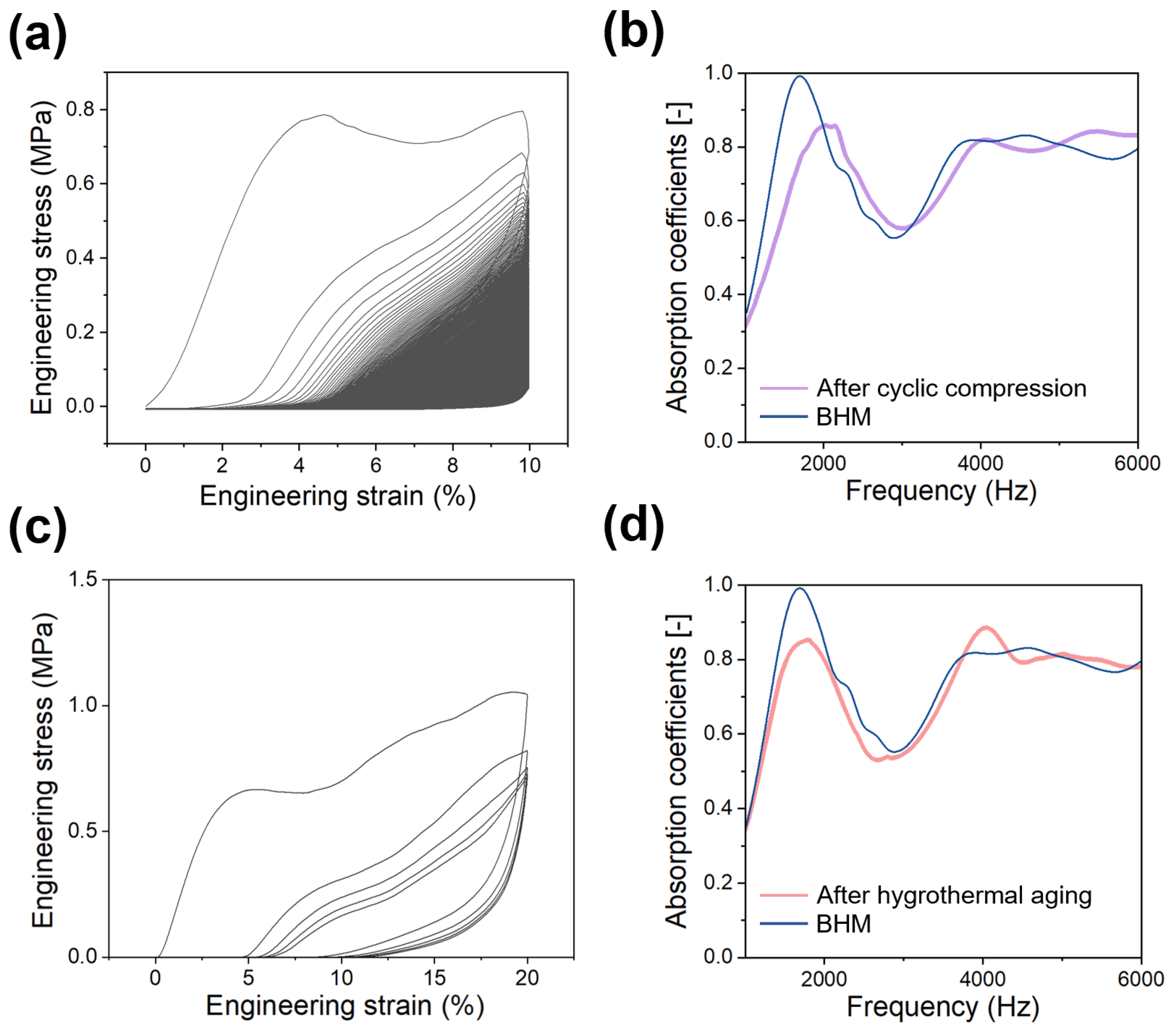
**

**Figure S19.** (a) Cyclic compression stress-strain curves of the BHM during 2000 loading–unloading cycles. (b) Absorption coefficient curves of the BHM after 2000 cycles. (c) Compression stress-strain curves of the BHM after 240 h of hygrothermal aging. (d) Absorption coefficient curves of the BHM after 240 h of hygrothermal aging.

**Table S1.** Comparison of broadband sound absorbers in terms of thickness, frequency range, and absorption performance.

| Absorber | Thickness  (mm) | Frequency range (Hz) | Average Sound absorption coefficient | Normalized average Sound absorption coefficient | Sound absorption coefficient  (f = 1870Hz) | Reference |
| --- | --- | --- | --- | --- | --- | --- |
| **BHM** | **22** | **1000-6000** | **0.742** | **0.034** | **0.95** | **This work** |
| Microlattice | 21 | 1000-6000 | 0.670 | 0.032 | 0.98 | ^[18]^ |
| Microlattice | 30 | 1000-6300 | 0.770 | 0.026 | 0.69 | ^[19]^ |
| Microlattice | 21 | 1000-6300 | 0.735 | 0.035 | 0.92 | ^[11]^ |
| Microlattice | 60 | 500-6000 | 0.600 | 0.010 | 0.43 | ^[20]^ |
| Microlattice | 24.2 | 1000-5000 | 0.600 | 0.025 | 0.80 | ^[21]^ |
| Microperforated panels | 24.5 | 50-5600 | 0.734 | 0.030 | 0.27 | ^[22]^ |
| Microperforated panels | 50 | 125-4000 | 0.626 | 0.013 | 0.81 | ^[23]^ |
| Microperforated panels | 20 | 125-4000 | 0.461 | 0.023 | 0.90 | ^[24]^ |
| Microperforated panels | 100 | 320-6400 | 0.930 | 0.009 | 0.95 | ^[25]^ |
| Aerogel | 25 | 100-6300 | 0.367 | 0.015 | 0.78 | ^[26]^ |
| Pectin cryogels | 20 | 500-6000 | 0.680 | 0.034 | 0.80 | ^[27]^ |

**Table S2.** Comparison of thermal conductivity, Young's modulus, and multifunctionality of various aerogel and foam materials.

| Materials | Thermal conductivity (mW·m⁻¹·K⁻¹) | Young's modulus (MPa) | Multifunctionality | Reference |
| --- | --- | --- | --- | --- |
| **Acrylate-based photopolymer** | **30.2** | **19.7** | **Sound absorption, Thermal insulation, Mechanical resilience** | **This work** |
| SiC aerogel | 21 | 24.4 | Impact resistance, Thermal insulation | ^[28]^ |
| SiO₂ ceramic aerogel | 32.6 | 21.79 | Thermal insulation | ^[29]^ |
| SiO₂@CNT | 31.2 | 14 | Thermal insulation | ^[30]^ |
| Alginate-based aerogel | 40 | 6.7 | Flame retardancy | ^[31]^ |
| SiC aerogel | 46 | 5.8 | Thermal insulation | ^[32]^ |
| Polyimide aerogel | 44 | 0.00712 | Water resistance,  Thermal insulation | ^[33]^ |
| Graphene/Al₂O₃ based aerogel | 50 | 0.255 | Thermal insulation | ^[34]^ |
| Carbon-tube based aerogel | 23 | 0.1 | Thermal insulation | ^[35]^ |
| Softwood foam | 31.1 | 0.13 | Thermal insulation | ^[36]^ |
| Softwood foam | 33.6 | 0.71 | Flame retardancy, Thermal insulation | ^[37]^ |
| Ultrahigh molecular weight PE film | 34 | 2.48 | Thermal insulation | ^[38]^ |

**S12.** **The explanation of design principles inspired by moth wings**

The ridge–cross rib architecture on the moth wing scale surface plays a crucial multifunctional role. Acoustically, these features reduce the effective bending stiffness, allowing for controllable local deformation and enhanced viscous and thermal dissipation. It has been reported that even with a wing thickness of only about 40 µm (approximately 1/100 of the incident wavelength), the moth wing can still achieve sound absorption as high as 72%.^[2]^ Compared with straight-walled or planar-cavity structures, the ridge–cross rib configuration introduced in our BHM elongates the effective cavity path and neck length while enlarging the equivalent volume within a thin and lightweight architecture, thereby maintaining excellent acoustic performance. Thermally, this structure features alternating chitin lamellae and air cavities, which enhance thermal insulation and temperature regulation in moths. By applying the same air-cavity principle, our BHM extends the heat transfer path, resulting in a low effective thermal conductivity. Mechanically, the directional alignment of scales and ridges imparts pronounced structural anisotropy, enabling efficient mechanical energy dissipation and superior impact resistance, which we have harnessed to improve the energy absorption capability of the BHM.

Inspired by these synergistic natural design principles, our BHM translates the graded porosity, structural anisotropy, and surface ridge architecture of moth wings into a controllable engineering framework, achieving broadband sound absorption, mechanical energy dissipation, and thermal insulation within a single thin and lightweight system. In contrast, conventional Helmholtz resonator arrays are typically designed for a single acoustic objective, often resulting in rigid structures with deep cavities that lack mechanical and thermal integration (Figure S20). Therefore, the moth-wing inspiration in our work goes far beyond the concept of a conventional resonator array and establishes a multifunctional and integrated design paradigm that enables the coupling of acoustic, mechanical, and thermal functionalities within a compact and lightweight structure. This design strategy also allows the BHM to be applied in scenarios where traditional Helmholtz resonators are unsuitable, such as protective helmets and UAV fuselages.

**
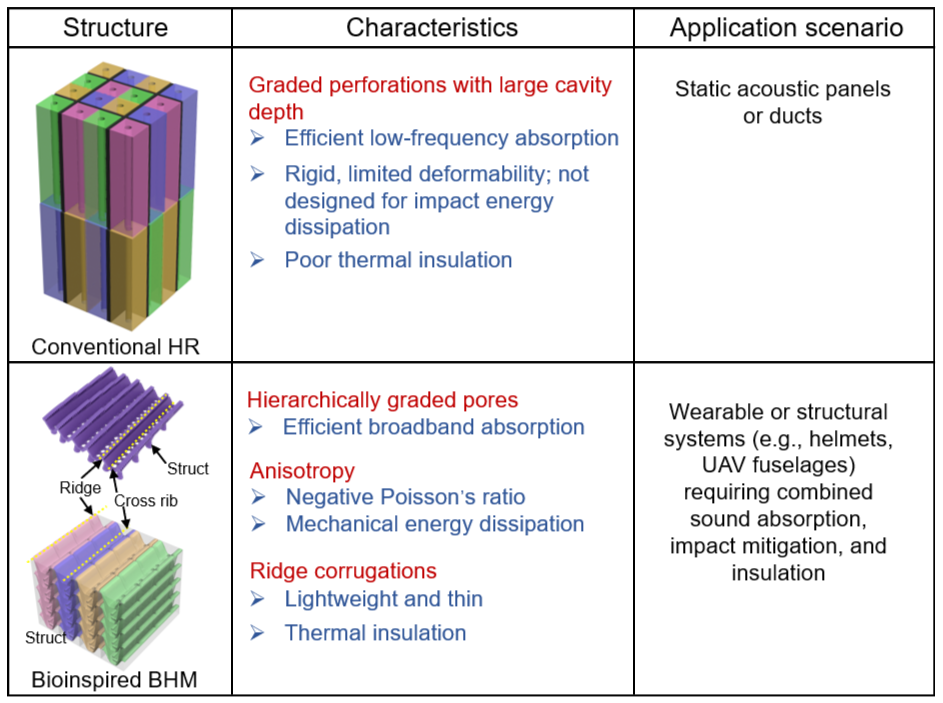
**

**Figure S20.** Structural and functional comparison of conventional Helmholtz resonator (HR) and bioinspired BHM.

**References**

[1] S. Ji, M. Qin, J. Zhao, H. Dai, J. Li, Biomimetic Water-Based Metamaterial Absorber for Ultrabroadband Radar Stealth, *Adv. Mater.* **2025**, e07439.

[2] T. R. Neil, Z. Shen, D. Robert, B. W. Drinkwater, M. W. Holderied, Moth Wings Are Acoustic Metamaterials, *Proc. Natl. Acad. Sci.* **2020**, *117*, 31134-31141.

[3] D. Balakrishnan, A. Prakash, B. J. Daurer, C. Finet, Y. C. Lim, Z. Shen, P. Thibault, A. Monteiro, N. Duane Loh, Nanoscale Cuticle Mass Density Variations Influenced by Pigmentation in Butterfly Wing Scales, *Nat. Commun.* **2025**, *16*, 7085.

[4] P. Kirya, A. Mestre-Farrera, J. Yang, L. V. Poulikakos, Bio-Inspired High-Temperature Superhydrophobic Coatings via Photocatalytic Spontaneous Co-Deposition, *Adv. Mater.* **2025**, *37*, 2407728.

[5] K. Krivoruchko, A. Goldshtein, A. Boonman, O. Eitan, J. Ben-Simon, V. D. Thong, Y. Yovel, Bats Evaluate Predator-Induced Risk Using Acoustically Eavesdropped Information From Prey, *iScience* **2021**, *24*, 102194.

[6] B. C. Leavell, J. J. Rubin, C. J. W. McClure, K. A. Miner, M. A. Branham, J. R. Barber, Fireflies Thwart Bat Attack With Multisensory Warnings, *Sci. Adv.* **2018**, *4*, eaat6601.

[7] A. Bay, P. Cloetens, H. Suhonen, J. P. Vigneron, Improved Light Extraction in the Bioluminescent Lantern of a *Photuris* Firefly, *Opt. Express* **2013**, *21*, 764–780.

[8] A. B. Kesel, Aerodynamic Characteristics of Dragonfly Wing Sections Compared With Technical Aerofoils, *J. Exp. Biol.* **2000**, *203*, 3125–3135.

[9] E. Appel, L. Heepe, C.-P. Lin, S. N. Gorb, Ultrastructure of Dragonfly Wing Veins: Composite Structure of Fibrous Material Supplemented by Resilin, *J. Anat.* **2015**, *227*, 561–582.

[10] S. R. Jongerius, D. Lentink, Structural Analysis of a Dragonfly Wing, *Exp. Mech.* **2010**, *50*, 1323–1334.

[11] X. Li, X. Yu, M. Zhao, Z. Li, Z. Wang, W. Zhai, Multi-Level Bioinspired Microlattice With Broadband Sound-Absorption Capabilities and Deformation-Tolerant Compressive Response, *Adv. Funct. Mater.* **2023**, *33*, 2210160.

[12] X. Li, X. Yu, J. W. Chua, W. Zhai, Harnessing Cavity Dissipation for Enhanced Sound Absorption in Helmholtz Resonance Metamaterials, *Mater. Horiz.* **2023**, *10*, 2892–2903.

[13] Y. Zhou, S. Shen, T. Liu, P. Li, F. Duan, Effective Heat Conduction Evaluation of Lattice Structures From Selective Laser Melting Printing, Int. J. *Heat Mass Transf.* **2024**, *218*, 124790

[14] M. Shahrzadi, M. Emami, A. H. Akbarzadeh, Heat Transfer in BCC Lattice Materials: Conduction, Convection, and Radiation, *Compos. Struct.* **2022**, *284*, 115159.

[15] X. Cheng, K. Wei, R. He, Y. Pei, D. Fang, The Equivalent Thermal Conductivity of Lattice Core Sandwich Structure: A Predictive Model, *Appl. Therm. Eng*. **2016**, *93*, 236-243.

[16] Z. Yang, Y. Bai, B. Wei, Y. Cui, J. Huang, Y. Li, L. Meng, Y. Wang, Durable Superhydrophobic–Oleophobic Polydimethylsiloxane-Based Coatings for Thermal Insulation, *Prog. Org. Coat.* **2023**, *174*, 107248.

[17] G. Primc, M. Mozetič, *Materials*, Surface Modification of Polymers by Plasma Treatment for Appropriate Adhesion of Coatings, **2024,** *17*, 1494.

[18] Z. Li, X. Wang, X. Li, Z. Wang, W. Zhai, New Class of Multifunctional Bioinspired Microlattice with Excellent Sound Absorption, Damage Tolerance, and High Specific Strength, ACS Appl. Mater. Interfaces **2023**, *15*, 9940-9952.

[19] X. Li, X. Yu, W. Zhai, Additively Manufactured Deformation-Recoverable and Broadband Sound-Absorbing Microlattice Inspired by the Concept of Traditional Perforated Panels, Adv. Mater. **2021**, *33*, 2104552.

[20] L. Chen, C. Wang, H. Ji, J. Qiu, Design and Analysis of a Microlattice Structure for Enhanced Broadband Sound Absorption, Applied Acoustics **2025**, *235*, 110681.

[21] Z. Liu, C. Dong, S. Li, X. Cong, X. Wang, C. Rudd, X. Yi, X. Liu, Novel design of skin Construction on the Sound Absorption of Honeycomb Sandwich Panels, Compos. Struct. **2025**, *356*, 118870.

[22] X. Li, S. Ding, X. Wang, S. L. A. Tan, W. Zhai, Recipe for Simultaneously Achieving Customizable Sound Absorption and Mechanical Properties in Lattice Structures, Adv. Mater. Technol. **2025**, *10*, 2400517.

[23] X. Li, X. Zhao, Y. Liu, A Resin-based Perforated Sound Absorption Composite Reinforced with Polyethylene Geotextile: Research on UV Resistance, Aging Resistance, Chemical Corrosion Resistance, Thermal Stability and Wear Resistance, Compos. Commun. **2025**, *58*, 102536.

[24] C. Dong, Z. Liu, R. Pierce, X. Liu, X. Yi, Sound absorption performance of A Micro Perforated Sandwich Panel with Honeycomb-Hierarchical Pore Structure Core, Applied Acoustics **2023**, *203*, 109200.

[25] Z. Zhou, S. Huang, D. Li, J. Zhu, Y. Li, Broadband Impedance Modulation via Non-Local Acoustic Metamaterials, Natl. Sci. Rev. **2022**, *9*, nwab171.

[26] Z. Zhao, H. Zhang, Q. Ma, J. He, M. Cheng, C. Long, H. Qi, Y. Cui, L. Pan, Resilient and Fatigue-Resistant Hybrid Fiber Aerogel with oriented Pore Structure for Broadband Frequency Sound Absorption, Compos. Sci. Technol. **2025**, *261*, 111004.

[27] F. Zou, J. Cucharero, Y. Dong, P. Kangas, Y. Zhu, J. Kaskirinne, G. C. Tewari, T. Hänninen, T. Lokki, H. Li, J. Vapaavuori, Maximizing Sound Absorption, Thermal Insulation, and Mechanical Strength of Anisotropic Pectin Cryogels, Chem. Eng. J. **2023**, *462*, 142236.

[28] L. Wang, J. Feng, Y. Luo, Y. Jiang, G. Zhang, J. Feng, Versatile Thermal-Solidifying Direct-Write Assembly Towards Heat-Resistant 3D-Printed Ceramic Aerogels for Thermal Insulation, *Small Methods* **2022**, *6*, 2200045.

[29] M. Miao, J. Yin, Z. Mao, Y. Chen, J. Lu, 3D-Printed Mullite-Reinforced SiC-Based Aerogel Composites, *Small* **2024**, *20*, 2401742.

[30] P. Guo, L. Su, K. Peng, D. Lu, L. Xu, M. Li, H. Wang, Additive Manufacturing of Resilient SiC Nanowire Aerogels, *ACS Nano* **2022**, *16*, 6625–6633.

[31] X. Zhao, K. Ruan, H. Qiu, X. Zhong, J. Gu, Fatigue-Resistant Polyimide Aerogels With Hierarchical Cellular Structure for Broadband Frequency Sound Absorption and Thermal Insulation, *Adv. Compos. Hybrid Mater.* **2023**, *6*, 171.

[32] Q. Zhang, D. Lin, B. Deng, X. Xu, Q. Nian, S. Jin, K. D. Leedy, H. Li, G. J. Cheng, Flyweight, Superelastic, Electrically Conductive, and Flame-Retardant 3D Multi-Nanolayer Graphene/Ceramic Metamaterial, *Adv. Mater.* **2017**, *29*, 1605506.

[33] Y. Shen, Z. Liu, G. Jiang, C. Li, Y. Guo, R. Chen, S. Guo, Fabrication of Light-Weight Ultrahigh Molecular Weight Polyethylene Films With Hybrid Porous Structure and the Thermal Insulation Properties, *J. Appl. Polym. Sci.* **2022**, *139*, e52403.

[34] Y. Zhu, I. Therrien, Z. Wan, Z. Yu, J. Zhu, D. Zheng, H. Sun, O. J. Rojas, F. Jiang, One-Pot Complexation of Phytic Acid and Polyethyleneimine on Cellulosic Microfibers Towards Insulative and Flame-Resistant Foam, *Int. J. Biol. Macromol.* **2024**, *275*, 133521.

[35] H.-J. Zhan, K.-J. Wu, Y.-L. Hu, J.-W. Liu, H. Li, X. Guo, J. Xu, Y. Yang, Z.-L. Yu, H.-L. Gao, X.-S. Luo, J.-F. Chen, Y. Ni, S.-H. Yu, Biomimetic Carbon Tube Aerogel Enables Super-Elasticity and Thermal Insulation, *Chem* **2019**, *5*, 1871–1882.

[36] Y. Zhu, J. Zhu, Z. Yu, Y. Ye, X. Sun, Y. Zhang, P. Zhu, F. Jiang, Air-Drying Scalable Production of Hydrophobic, Mechanically Stable, and Thermally Insulating Lignocellulosic Foam, *Chem. Eng. J.* **2022**, *450*, 138300.

[37] R. Zhang, Y. Fu, W. Qin, S. Qiu, J. Chang, Preparation of a Novel Bio-Based Aerogel With Excellent Hydrophobic Flame-Retardancy and High Thermal Insulation Performance, *J. Appl. Polym. Sci.* **2024**, *141*, e55416.

[38] A. Lamy-Mendes, W. J. Malfait, A. Sadeghpour, A. V. Girão, R. F. Silva, L. Durães, Influence of 1D and 2D Carbon Nanostructures in Silica-Based Aerogels, *Carbon* **2021**, *180*, 146–162.
